# Hawking-Type Singularity Theorems for Worldvolume Energy Inequalities

**DOI:** 10.1007/s00023-024-01502-6

**Published:** 2024-11-25

**Authors:** Melanie Graf, Eleni-Alexandra Kontou, Argam Ohanyan, Benedict Schinnerl

**Affiliations:** 1 Faculty of Mathematics, University of Tübingen, Auf der Morgenstelle 10, 72076 Tübingen, Germany; 2 ITFA and GRAPPA, Universiteit van Amsterdam, Science Park 904, Amsterdam, The Netherlands; 3 Department of Mathematics, King’s College London, Strand, London, WC2R 2LS UK; 4 Faculty of Mathematics, University of Vienna, Oskar-Morgenstern Platz 1, 1090 Vienna, Austria

**Keywords:** 83C75, 53C50, 53B30, 70S20

## Abstract

The classical singularity theorems of R. Penrose and S. Hawking from the 1960s show that, given a pointwise energy condition (and some causality as well as initial assumptions), spacetimes cannot be geodesically complete. Despite their great success, the theorems leave room for physically relevant improvements, especially regarding the classical energy conditions as essentially any quantum field theory necessarily violates them. While singularity theorems with weakened energy conditions exist for worldline integral bounds, so-called worldvolume bounds are in some cases more applicable than the worldline ones, such as the case of some massive free fields. In this paper, we study integral Ricci curvature bounds based on worldvolume quantum strong energy inequalities. Under the additional assumption of a—potentially very negative—global timelike Ricci curvature bound, a Hawking-type singularity theorem is proved. Finally, we apply the theorem to a cosmological scenario proving past geodesic incompleteness in cases where the worldline theorem was inconclusive.

## Introduction

The singularity theorems of Penrose [30] and Hawking [24] represent an important breakthrough in classical general relativity. They showed that gravitational systems will originate or terminate in a singularity, defined in that context as geodesic incompleteness, under general conditions. For a recent review article on singularity theorems, see [33].

Both theorems, along with mild causality conditions, require the non-negativity of contractions of the Ricci tensor. These conditions are assumed to represent properties of the matter content of the spacetime since, with the use of the Einstein Equation, they can be re-written as energy conditions, i.e., restrictions on contractions of the stress energy tensor. The Hawking singularity theorem uses the timelike convergence condition

1 $$\begin{aligned} R_{\mu \nu }U^\mu U^\nu \ge 0 , \end{aligned}$$

where $U^\mu$ is a timelike vector. With the use of the Einstein equation, it becomes the strong energy condition (SEC)

2 $$\begin{aligned} \rho _U \equiv \left( T_{\mu \nu } -\frac{T g_{\mu \nu }}{n-2} \right) U^\mu U^\nu \ge 0 , \end{aligned}$$

where *n* is the spacetime dimension and *T* the trace of the stress energy tensor. The quantity on the left is the effective energy density (EED), a term introduced in [4]. The SEC has an unclear physical interpretation [7, 26] and is easily violated by classical fields, for example, the free massive Klein–Gordon field. The situation is even worse when including quantum effects. It has long been known [8] that quantum fields can admit negative energies violating all pointwise energy conditions. Average energy conditions and quantum energy inequalities (QEIs) are weaker restrictions on the energy density (and similar quantities) or its renormalized expectation value in the case of quantum fields. Starting from the seminal work of Ford [17], QEIs have been derived for flat and curved spacetimes and a variety of mainly free fields (see Refs. [9, 26] and references within). They often take the form of

3 $$\begin{aligned} \langle \rho (f) \rangle _\omega \ge -|||f||| , \end{aligned}$$

where *f* is a smooth compactly supported real-valued function. Here $\rho$ is the renormalized energy density, $\omega$ is a Hadamard state, and $||| \cdot |||$ is a Sobolev norm. The integral is more often over a timelike geodesic, but it can also be over a worldvolume.

With the use of the classical or the semiclassical Einstein equation, average energy conditions and QEIs become conditions on the Ricci tensor and may be used as alternative assumptions for singularity theorems. Starting with the work of Tipler [36], various authors have proved singularity theorems with weaker energy conditions [3, 31]. First, Fewster and Galloway [10] proved singularity theorems with a condition of the form of (3) using the Raychaudhuri equation. More recently, Fewster and Kontou [12] presented proofs with a similar energy condition using index form methods and estimated the required initial contraction or mean normal curvature of a Cauchy surface.

All of these works use energy conditions averaged over a single timelike or null geodesic. However, there is strong motivation to prove singularity theorems using worldvolume bounds instead. In the timelike case, the worldvolume bounds are often stronger than the worldline ones for both classical [4] and quantum fields [11]. In particular, in the case of a massive scalar, the mass dependence can be removed in the case of worldvolume bounds.

More important motivation to consider worldvolume inequalities arises in the null case. Fewster and Roman showed with an explicit counterexample [14] that QEIs over a null geodesic segment do not admit finite lower bounds. However, this is not the case if the integral is over two null directions as it was recently proven [15]. Using such an inequality as an assumption to a singularity theorem would lead to the proof of a semiclassical singularity theorem for null geodesic incompleteness. Previously, only two semiclassical singularity theorems have been proven: one for the timelike incompleteness case [13] and one for the null incompleteness case [18], but the latter required the introduction of a theory-dependent UV cutoff significantly weakening the result.

The consequence of the classical singularity theorems is the existence of an incomplete causal geodesic. If one could simply extend the spacetime to a complete one, this would in some sense signify that the incompleteness was not physically reasonable as there would be another better suited spacetime to model the situation. Of course, the singularity theorems also imply that one cannot accomplish this with a $C^2$-Lorentzian metric without violating either the energy or causality condition.

Therefore, the question arises whether one could instead resolve this issue by extending with a lower regularity Lorentzian metric, i.e., given a $C^2$-spacetime satisfying the assumptions of the singularity theorems, could there be a (timelike and/or null geodesically) complete low-regularity extension of it, satisfying appropriate generalizations of the assumptions? In other words, could the singularity predicted by the smooth theorems merely be due to a drop in regularity (which could be akin to a jump or delta distribution in the curvature)?

This question is essentially equivalent to whether singularity theorems continue to hold for lower regularity metrics which was already raised and extensively discussed in [25, Sec. 8.4]. Nevertheless, it has only recently become rigorously tractable mathematically (cf.[21, 22, 27–29, 32]) and trying to resolve it fully continues to be an active pursuit of major physical importance because a plethora of physical models involve spacetime metrics which are not $C^2$ (e.g., the Oppenheimer–Snyder model of a collapsing star, matched spacetimes, gravitational shock waves, thin mass shells, ...). For a recent comprehensive review of this research area, see [35].

The current proofs of low-regularity singularity theorems commonly employ an approximation-based approach aiming to distill and exploit some stability properties of both the conditions in the singularity theorems and the formation or non-formation of singularities. Note, however, that already within the class of smooth metrics, Ricci curvature being nonnegative along timelike or null geodesics is unstable: Even if a metric $g_0$ satisfies the timelike or null convergence condition, metrics $g_\varepsilon$ converging to $g_0$ will in general violate these conditions by some small error, with the exact nature of the introduced error depending on the topology of the convergence. If $g_0$ is a non-smooth metric satisfying only a distributional version of the strong or null energy condition, then the possible topologies of convergence and therefore the form of the energy condition recovered for $g_\varepsilon$ are limited by the initial regularity of $g_0$. The current state of the art regarding low-regularity singularity theorems [21, 27] considers $g_0\in C^{1}$. It was shown that in this case—for a family of approximations $g_\varepsilon$ constructed in a very specific way— convergence is strong enough, but only barely, to recover a classical energy condition for the approximating $g_\varepsilon$ up to “pointwise small” violations. That is, one obtains a condition of the form $\textrm{Ric}[g_\varepsilon ](X,X) \ge -\delta _\varepsilon$ for all $g_\varepsilon$-timelike/null vectors *X* contained in some compact subset $K\subseteq TM$, where $0<\delta _\varepsilon \equiv \delta _\varepsilon (K) \rightarrow 0$. Such an energy condition is still amenable to classical Riccati or index form techniques to show that (for small enough $\delta _\varepsilon$ and large enough *K* depending on the given initial condition) certain timelike geodesics must encounter focal points before a fixed time which then can be leveraged to obtain that, in the limit, a singularity must exist for the original metric. However, if one were only to obtain weaker convergence, one expects that such pointwise estimates cannot be recovered from the distributional energy conditions placed on $g_0$ and that one instead will have to make do with integral bounds of some form, such as the $L^1$-bound we study in Theorem 4.1.

In this work, we will address the issues arising from starting with a worldvolume bound by translating these bounds into bounds along single timelike geodesics by using ideas inspired by the so-called segment inequality from Riemannian geometry due to Cheeger–Colding [5] as well as an $L^1$-Ricci bound-based Myers theorem by Sprouse [34]. More specifically, we consider volume integral bounds of the form

4 $$\begin{aligned} \frac{1}{\sigma (B)} \int _{\Omega _T^+(B)} |{{\,\textrm{Ric}\,}}(U_p,U_p)_-| \, dvol_g(p) \le C \end{aligned}$$

for suitable subsets *B* of a Cauchy surface $\Sigma$ in *M* (here *C* is a constant, $U_p$ is the vector field that is tangent to normal geodesics from $\Sigma$ at *p*, $\sigma$ is the intrinsic Riemannian volume measure of $\Sigma$, and $\Omega _T^+(B)$ are those points that can be reached from *B* when evolving until time *T*), as well as

5 $$\begin{aligned} \int _M {{\,\textrm{Ric}\,}}(U_p,U_p) F(p)^2 \, dvol_g(p) \ge -Q_1 \Vert F\Vert _{L^2}^2 - Q_2 \Vert \dot{F}\Vert _{L^2}^2\quad \quad \forall F\in C^\infty _c(\mathcal {M}^+), \end{aligned}$$

where $Q_1,Q_2$ are suitable constants and $\mathcal {M}^+$ is the cut point free future evolution of $\Sigma$, see Sect. 2 for definitions and Sect. 4 for precise statements of our results. We then reduce these inequalities to inequalities along single timelike geodesics: By standard results from Lorentzian geometry, the normal exponential map provides a diffeomorphism from a neighborhood of the normal bundle of $\Sigma$ to a neighborhood of $\Sigma$. In such a region, the evolution of the surface is well-defined and one can use its geometric properties (like the infinitesimal area element and the mean curvature). However, as soon as the normal geodesics to $\Sigma$ encounter cut points, the evolution surfaces will degenerate or form caustics. Hence, we will have to restrict our analysis to regions where $\Sigma$ has a well defined evolution. This will in turn allow us, using exponential coordinates, to naturally split a volume integral into an iterated integral along geodesics of the congruence and over a subset of $\Sigma$. However, the volume element will not be constant in time in these coordinates so the integrand in each line integral will not only involve the Ricci curvature along the geodesic but an additional geometric factor determined by the time evolution of the area element of $\Sigma$. To be able to deal with this additional factor, we need to develop new estimates on the time evolution of the area element from *below* resp. *backwards in time*. From there on, we are able to extract bounds on a single line integral and use classical index form methods to deduce geodesic focusing given an appropriate initial mean curvature condition on $\Sigma$.

This paper is organized as follows. In Sect. 2, we give a series of definitions and known results on spacetime geometry that will be needed later. In Sect. 3, we provide area comparison results that are used to derive bounds on integrals along geodesics from worldvolume integrals. In Sect. 4, we present the main result of this paper, a singularity theorem with a worldvolume integrated energy condition as an assumption. In Sect. 5, we present a physical application of our theorem for the case of the classical non-minimally coupled scalar field. In Sect. 6, we apply the theorem in the case of a simple cosmological model proving past timelike geodesic incompleteness for a few different fields. We conclude in Sect. 7 with a summary and discussion of future work.

### Notation and Conventions

Let us collect some remarks on the notation and conventions that we will use throughout. First, a spacetime is a pair (*M*, *g*) where *M* is a 2nd countable, Hausdorff smooth manifold, and *g* is a (smooth) metric on *M* with signature $(-,+,\dots ,+)$. The d’Alembertian operator with respect to the metric *g* is defined as $\Box _g:= -g^{\mu \nu } \nabla _\mu \nabla _\nu$. Note that this sign convention for $\Box _g$ is consistent with the physics literature references of Sects. 5–6, but differs from the one used in most of the references for Sects. 2, 4. The Riemann curvature tensor of *g* is $R(X,Y)Z = \nabla _X \nabla _Y Z - \nabla _Y \nabla _X Z - \nabla _{[X,Y]}Z$, and the Ricci tensor ${{\,\textrm{Ric}\,}}$ is its (1, 3)-contraction. For $A \subseteq M$, we write $\tau _A$ for the (forward) time separation to *A*, i.e. $\tau _A(p):=\sup _{q \in A} \tau (q,p)$. A Cauchy surface in a spacetime is a set which is uniquely met by each inextendible causal curve. Given a spacelike hypersurface $\Sigma$ of a spacetime (*M*, *g*), the second fundamental form is the map $II:\mathfrak {X}(\Sigma ) \times \mathfrak {X}(\Sigma ) \rightarrow \mathfrak {X}(\Sigma )^{\perp }$, $II(X,Y):=(\nabla _X Y)^{\perp }$, where $\nabla$ is the Levi-Civita connection in *M*. For the mean curvature of a spacelike hypersurface, we adopt the conventions in [38], i.e., if $\textbf{n}$ is the future unit normal to the hypersurface, then the mean curvature is given by $H = \textrm{tr}(\nabla \textbf{n})$.

We use geometric units throughout this work (i.e. $G=c=1$) except for Sect. 6. Let us note that we will employ index notation in Sect. 5, as is customary in the physical literature.

## Geometric Background

In this section, we collect several well-known results from spacetime geometry which will be needed for what follows. Throughout, let (*M*, *g*) be a smooth spacetime of dimension *n* and let $\Sigma \subseteq M$ be a smooth, spacelike Cauchy surface with future timelike unit normal $\textbf{n}$. We will often only consider future versions in definitions and results; past versions are obtained by duality.

As we will see in this section, the exponential map not only yields a diffeomorphism from a neighborhood of its normal bundle to a neighborhood of our spacelike Cauchy surface $\Sigma$, it also provides us with a foliation of that domain by hypersurfaces perpendicular to the congruence of normal vector fields emanating from $\Sigma .$ This is guaranteed as long as the geodesic congruence does not encounter cut points, as then the exponential map no longer is a diffeomorphism. We will mostly reference [37] for proofs of the results presented in this section, for an alternative textbook reference see [2, Sect. 9].

Since we will have to deal with the case where *M* is not future geodesically complete, let $[0,s^+(\textbf{x}))$ denote the maximal interval of definition for the unique geodesic $\gamma$ with initial data $\gamma (0)=\textbf{x}\in \Sigma$, $\dot{\gamma }(0)=\textbf{n}_\textbf{x}$.

Note that the future normal bundle of $\Sigma$ is of the form $N^+\Sigma =\{t\textbf{n}_\textbf{x}: (t,\textbf{x}) \in [0,\infty )\times \Sigma \} \cong [0,\infty ) \times \Sigma$. Let $\mathcal {I}^+:=\{(t,\textbf{x}) \in [0,\infty ) \times \Sigma : t\in [0,s^+(\textbf{x})) \}\subseteq [0,\infty )\times \Sigma$.

### Definition 2.1

*(Future normal exponential map).* The *future normal exponential map* of $\Sigma$ is the map $\exp _{\Sigma }^+:\mathcal {I}^+ \rightarrow M$ defined by

6 $$\begin{aligned} \exp _{\Sigma }^+(t,\textbf{x}):=\exp _{\textbf{x}}(t \textbf{n}_{\textbf{x}}). \end{aligned}$$

Let us now recall the definition of (future) cut points and the (future) cut function.

### Definition 2.2

*(Future cut function and future cut points).* The *future cut function* $c_{\Sigma }^+:\Sigma \rightarrow (0,\infty ]$ of $\Sigma$ is defined via

7 $$\begin{aligned} c_{\Sigma }^+(\textbf{x}):= \sup \{t \in [0,s^+(\textbf{x})) \mid \tau _{\Sigma }(\exp _{\textbf{x}}(t \textbf{n}_{\textbf{x}})) = t\}. \end{aligned}$$

If $c_{\Sigma }^+(\textbf{x}) < s^+(\textbf{x})$ for some $\textbf{x}\in \Sigma$, we call the point $\exp _{\textbf{x}}(c_{\Sigma }^+(\textbf{x}) \textbf{n}_{\textbf{x}})$ a *future cut point* of $\Sigma$. We write ${{\,\textrm{Cut}\,}}^+(\Sigma )$ for the set of future cut points of $\Sigma$.

### Lemma 2.3

The future cut function $c_{\Sigma }^+$ is lower semicontinuous and satisfies $c_{\Sigma }^+(\textbf{x}) > 0$ for all $\textbf{x}\in \Sigma$. Moreover, ${{\,\textrm{Cut}\,}}^+(\Sigma ) \subseteq M$ is a set of measure zero with respect to the volume measure.

### Proof

Lower semicontinuity follows from [37, Prop.3.2.29]. Since any geodesic normal to $\Sigma$ must maximize initially (cf. [37, Cor. 3.2.23]), it follows that $c_{\Sigma }^+(\textbf{x}) > 0$ for all $\textbf{x}\in \Sigma$. The claim about the measure of ${{\,\textrm{Cut}\,}}^+(\Sigma )$ is proved in [37, Prop. 3.2.32]. $\square$

### Lemma 2.4

The future normal exponential map $\exp _{\Sigma }^+$ is a diffeomorphism (of manifolds with boundary) from a neighborhood $\mathcal {D}^+$ of $\{0\} \times \Sigma$ onto $\mathcal {M}^+:=J^+(\Sigma ) \setminus {{\,\textrm{Cut}\,}}^+(\Sigma )$.

### Proof

This follows from [37, Thm. 3.2.31]. $\square$

### Remark 2.5

As shown in the reference given above, the set $\mathcal {D}^+$ is explicitly given by

8 $$\begin{aligned} \mathcal {D}^+ = \{(t,\textbf{x}) \in [0,\infty ) \times \Sigma \mid \textbf{x}\in \Sigma , t \in [0,c_{\Sigma }^+(\textbf{x}))\}^\circ . \end{aligned}$$

### Lemma 2.6

Consider the diffeomorphism $\exp _{\Sigma }^+: \mathcal {D}^+ \rightarrow \mathcal {M}^+$. Then, for any $t\ge 0$ the sets $(\{t\} \times \Sigma ) \cap \mathcal {D}^+ \subseteq \mathcal {D}^+$ and for any $t\ge 0$, $\mathcal {S}_t:=\{\tau _{\Sigma } = t\} \cap \mathcal {M}^+ \subseteq \mathcal {M}^+$ are either empty or embedded hypersurfaces (in the respective supersets). Moreover, if nonempty, $\mathcal {S}_t$ is spacelike and the restriction of the exponential map $\exp _{\Sigma }^+: (\{t\} \times \Sigma ) \cap \mathcal {D}^+ \rightarrow \mathcal {S}_t$ is a diffeomorphism.

### Proof

Since $\tau$ is smooth on $\mathcal {M}^+$ with timelike gradient by [37, Prop. 3.2.33], $\mathcal {S}_t$ is an embedded, spacelike hypersurface.

Since $\exp _{\Sigma }^+: \mathcal {D}^+ \rightarrow \mathcal {M}^+$ is a diffeomorphism, for any $q\in \mathcal {S}_t \subseteq \mathcal {M}^+$ there exists a point $(t',\textbf{x})\in \mathcal {D}^+$ with $\exp _{\Sigma }^+(t',\textbf{x})=q$, so by Rem. 2.5, $t'<c_\Sigma ^+(\textbf{x})$ so $s\mapsto \exp _{\Sigma }^+(s,\textbf{x})$ is maximizing until parameter $t'$ and hence $t'=\tau _{\Sigma }(q)=t$. So $\mathcal {S}_t\subseteq \exp _\Sigma ^+((\{t\} \times \Sigma )\cap \mathcal {D}^+)$. On the other hand, any $(t,\textbf{x})\in \mathcal {D}^+$ satisfies $t<c_\Sigma ^+(\textbf{x})$ by Rem. 2.5 and hence $\tau _{\Sigma }(\exp _{\Sigma }^+(t,\textbf{x})) =t$. So the restriction of $\exp _\Sigma ^+$ to $(\{t\} \times \Sigma )\cap \mathcal {D}^+$ is bijective. It is a diffeomorphism because $\exp _\Sigma ^+:\mathcal {D}^+ \rightarrow \mathcal {M}^+$ is one. $\square$

### Lemma 2.7

Consider the diffeomorphism $\exp _{\Sigma }^+:\mathcal {D}^+ \rightarrow \mathcal {M}^+$. Then the pullback measure on $\mathcal {D}^+$ of the volume measure $vol_g$ on $\mathcal {M}^+$ under this diffeomorphism (i.e., the standard pushforward measure under its inverse) is of the form

9 $$\begin{aligned} \mathcal {A} \, dt \otimes d\sigma , \end{aligned}$$

where $\sigma$ is the volume measure on $\Sigma$ with respect to the Riemannian metric induced by *g*. The infinitesimal area $\mathcal {A} = \mathcal {A}(t,\textbf{x}):\mathcal {D}^+\rightarrow (0,\infty )$ is smooth in $\textbf{x}$ and

10 $$\begin{aligned} \int _{\textrm{pr}_\Sigma ((\exp _\Sigma ^+)^{-1}(\mathcal {S}_t))} \mathcal {A}(t,\textbf{x}) d\sigma (\textbf{x}) = \sigma _t(\mathcal {S}_t), \end{aligned}$$

where $\sigma _t$ is the Riemannian measure on $\mathcal {S}_t$. In particular, $\mathcal {A}(0,\textbf{x}) = 1$ for all $\textbf{x}\in \Sigma$.

### Proof

This is basically the “measure version” of the coarea formula, cf. [38, Prop. 3]. The coarea formula implies that for any $f \in L^1(J^+(\Sigma ))$,

11 $$\begin{aligned} \int _{J^+(\Sigma )} f \, dvol_g = \int _{\mathcal {M}^+} f \, dvol_g = \int _0^{\infty } \int _{\mathcal {S}_t} f|_{\mathcal {S}_t} d\sigma _t \, dt. \end{aligned}$$

By definition of the pushforward of a measure, we get that

12 $$\begin{aligned} \int _{\mathcal {S}_t} f|_{\mathcal {S}_t} \, d\sigma _t&= \int _{\mathcal {S}_t} (f \circ \exp _{\Sigma }^+) \circ (\exp _{\Sigma }^+)^{-1} \, d\sigma _t \nonumber \\&= \int _{(\{t\} \times \Sigma ) \cap \mathcal {D}^+} (f \circ \exp _{\Sigma }^+) \, d((\exp _{\Sigma }^+)^{-1}_* \sigma _t). \end{aligned}$$

Since the volume form is locally an *n*-fold wedge, the pushforward of $\sigma _t$ under $(\exp _{\Sigma }^+)^{-1}$ is proportional to the Riemannian measure on $(\{t\} \times \Sigma ) \cap \mathcal {D}^+$ (which by identification is just $\sigma$), so there is a smooth (in $\textbf{x}\in \Sigma$) function $\mathcal {A}(t,\textbf{x})$ such that $d((\exp ^{+})^{-1}_* \sigma _t) = \mathcal {A}(t,.) \,d\sigma$. Reinserting this into the previous calculation, and then that back into the coarea formula, we get

13 $$\begin{aligned} \int _{\mathcal {M}^+} f \, dvol_g = \int _0^{\infty } \int _{(\{t\} \times \Sigma ) \cap \mathcal {D}^+} (f \circ \exp _{\Sigma }^+) \mathcal {A}\, d\sigma dt = \int _{\mathcal {D}^+} (f \circ \exp _{\Sigma }^+) \mathcal {A} \, dt\otimes d\sigma . \end{aligned}$$

Since this holds for all integrable *f*, the claim about the measures follows. $\square$

Let us recapitulate: For any $f \in L^1(J^+(\Sigma ))$, we have

14 $$\begin{aligned} \int _{\mathcal {M}^+} f \, dvol_g = \int _{\mathcal {D}^+} (f \circ \exp _{\Sigma }^+) \, \mathcal {A} \, dt \otimes d\sigma . \end{aligned}$$

The same relation is true if we integrate over any measurable subset $C \subseteq \mathcal {D}^+$ and $\exp _{\Sigma }^+(C) \subseteq \mathcal {M}^+$.

### Remark 2.8

If we write $g(t,\textbf{x}) = g(\exp _{\Sigma }^+(t,\textbf{x}))$, then, upon choosing a chart in $\Sigma$, it follows that $\mathcal {A}(t,\textbf{x}) = \frac{\sqrt{\det g(t,\textbf{x})}}{\sqrt{\det g(0,\textbf{x})}}$, in particular $\mathcal {A}$ is smooth in both variables. This can be seen from the splitting of the metric in $\exp _{\Sigma }^+$-coordinates, i.e.,

15 $$\begin{aligned} (\exp _{\Sigma }^+)^* g = -dt^2 + g_t, \end{aligned}$$

where $g_t$ is the Riemannian metric induced by *g* on $\mathcal {S}_t$. Later, we will be interested in proving comparison results for the infinitesimal area $\mathcal {A}(t,\textbf{x})$, which then directly imply more or less identical results for $\sqrt{\det g(t,\textbf{x})}$.

We are interested in regions of $\Sigma$ such that the normal geodesics emanating from there are free of cut points up to a certain length. Working with such regions, rather than $\Sigma$, will allow us to use the normal exponential map in our integral calculations.

### Definition 2.9

*(Future evolutions, regular points).*

1. For $T > 0$ and $B \subseteq \Sigma$, define the *future evolution* of *B* up to *T* via
  16 $$\begin{aligned} \Omega _T^+(B):=\{\exp _{\textbf{x}}(t\textbf{n}_{\textbf{x}}): \textbf{x}\in B, t \in [0,T]\cap [0,c_\Sigma ^+(\textbf{x}))\}. \end{aligned}$$
2. For $T,\eta > 0$, the *set of *$T+\eta$*-regular points* ${{\,\mathrm{Reg^+_{\eta }}\,}}(T) \subseteq \Sigma$ is defined to be the set of all $\textbf{x}\in \Sigma$ such that $s^+(\textbf{x})>T+\eta$ and $\exp _{\Sigma }^+(.,\textbf{x}):[0,T+\eta ] \rightarrow M$ is $\Sigma$-maximizing.

Usually, $\eta$ will be much smaller than *T* and is there to guarantee a lack of cut points for a short time beyond *T*.

### Remark 2.10

(On $$\Omega _T^+$$, $${{\,\mathrm{Reg^+_{\eta }}\,}}(T)$$).

1. By definition, ${{\,\mathrm{Reg^+_{\eta }}\,}}(T) = (c_{\Sigma }^+)^{-1}([T+\eta ,\infty ])$ so ${{\,\mathrm{Reg^+_{\eta }}\,}}(T)$ is measurable by the lower semicontinuity of $c_{\Sigma }^+$.
2. It is easy to see that for any $B \subseteq {{\,\mathrm{Reg^+_{\eta }}\,}}(T)$,
  17 $$\begin{aligned} \mathcal {M}^+ \supseteq \Omega _T^+(B) = \exp _{\Sigma }^+ ([0, T] \times B \}). \end{aligned}$$

### Definition 2.11

*(Congruence of* $\Sigma$). Consider the diffeomorphism $\exp _{\Sigma }^+:\mathcal {D}^+ \rightarrow \mathcal {M}^+$. Let $Y \in \mathfrak {X}({{\,\mathrm{\mathbb {R}}\,}}\times \Sigma )$ denote the canonical vector field $Y:(t,\textbf{x}) \mapsto (t,0) \in {{\,\mathrm{\mathbb {R}}\,}}\times T_\textbf{x}\Sigma$. Now consider the restriction of *Y* as a vector field on $\mathfrak {\mathcal {D}^+}$ and let $U \in \mathfrak {X}(\mathcal {M}^+)$ denote its pushforward under the diffeomorphism $\exp _{\Sigma }^+$. We call *U* the *future unit congruence (vector field)* of $\Sigma$. Explicitly, if $p = \exp _{\Sigma }^+(t,\textbf{x}) \in \mathcal {M}^+$, then

18 $$\begin{aligned} U(p) = \frac{d}{ds}\Big |_{s=0} \exp _{\textbf{x}}((t+s)\textbf{n}_{\textbf{x}}), \end{aligned}$$

i.e., *U* assigns to $p \in \mathcal {M}^+$ the tangent vector (at *p*) of the unique normal unit speed geodesic from $\Sigma$ that passes through *p*.

## Forward and Backward Comparison Results

In the following, we discuss comparison results for the infinitesimal area $\mathcal {A}(t,\textbf{x})$ as well as the d’Alembert operator of the (signed) time separation. As they will be used in various estimates, we give a detailed treatment of all the comparison constants.

In addition to the assumptions from the previous section, we assume that (*M*, *g*) satisfies ${{\,\textrm{Ric}\,}}_g(X,X) \ge n\kappa$ for all unit timelike vectors *X* and some $\kappa <0$. Further, the mean curvature *H* of $\Sigma$ with respect to the future normal $\textbf{n}$ satisfies $H \le \beta < 0$.

We start by proving an infinitesimal version of an area comparison result given in [38, Thm. 8], which allows for a direct estimation of the time evolution of the area element by the modified distance functions of constant curvature spaces.

### Remark 3.1

(Comparison geometries).

For completeness, we briefly summarize the model warped product geometries $$(a,b)\times _f (\Sigma ,h)$$ with $$(\Sigma ,h)$$ a complete Riemannian manifold and Lorentzian metric $$g= -dt^2+f^2 h $$ from [38, Sec. 4.2]. We want the model geometries to have constant Ricci curvature $${{\,\textrm{Ric}\,}}_{g} = -n \kappa g$$. We further require the mean curvature of the $$\{t=0\}$$-slice in the model geometry to equal $$\beta $$; hence, we have a two-parameter family of model geometries and will henceforth index all quantities in the model geometries with $$\kappa $$ and $$\beta $$. As was argued in [38], these requirements force the Riemannian factor to be Einstein and (up to rescaling) uniquely determine $$f_{\kappa ,\beta }$$. Tables containing a complete list of all possible comparison warping functions $$f_{\kappa ,\beta }$$ are found, e.g., in [38] Table 1 or [20] Table 1. Congruent with our assumptions, we will only elaborate on the case $$\kappa <0$$ and $$\beta <0$$ here, distinguishing three different cases:

1. $\beta > -(n-1) \sqrt{|\kappa |}$, where we can take $(\Sigma _{\kappa ,\beta },h_{\kappa ,\beta })$ to be the $(n-1)$-dimensional unit sphere, in which case $a_{\kappa ,\beta }=-\infty$ and $b_{\kappa ,\beta }=\infty$,
  19 $$\begin{aligned} f_{\kappa ,\beta }(t) = \frac{1}{\sqrt{|\kappa |}} \cosh \left( \sqrt{|\kappa |}t + \text {artanh}\left( \frac{\beta }{(n-1)\sqrt{|\kappa |}}\right) \right) \end{aligned}$$
  In this case, the mean curvature of the hypersurface $\{t\}\times \Sigma _{\kappa ,\beta }$, $H_{\kappa ,\beta }(t)$, is given by
  20 $$\begin{aligned} H_{\kappa ,\beta }(t)= &   (n-1) \frac{f_{\kappa ,\beta }'(t)}{f_{\kappa ,\beta }(t)}\nonumber \\= &   (n-1) \sqrt{|\kappa |}\tanh \left( \sqrt{|\kappa |}t + \text {artanh}\left( \frac{\beta }{(n-1)\sqrt{|\kappa |}}\right) \right) \end{aligned}$$
2. $\beta = -(n-1) \sqrt{|\kappa |}$, where we can take $(\Sigma _{\kappa ,\beta },h_{\kappa ,\beta })$ to be flat ${{\,\mathrm{\mathbb {R}}\,}}^{n-1}$, in which case $a_{\kappa ,\beta }=-\infty$ and $b_{\kappa ,\beta }=\infty$ and
  21 $$\begin{aligned} f_{\kappa ,\beta }(t) = \exp (-\sqrt{|\kappa |}t) \end{aligned}$$
3. $\beta < -(n-1) \sqrt{|\kappa |}$, where we can take $(\Sigma _{\kappa ,\beta },h_{\kappa ,\beta })$ to be unit $(n-1)$-dimensional hyperbolic space, in which case $a_{\kappa ,\beta }=-\infty$ and $b_{\kappa ,\beta }= - \frac{1}{\sqrt{|\kappa |}} \text {arcoth}\left( \frac{\beta }{(n-1)\sqrt{|\kappa |}}\right)$ (i.e., the model geometries are future timelike goedesically incomplete),
  22 $$\begin{aligned} f_{\kappa ,\beta }(t) = \frac{1}{\sqrt{|\kappa |}} \sinh \left( \sqrt{|\kappa |}t + \text {arcoth}\left( \frac{\beta }{(n-1)\sqrt{|\kappa |}}\right) \right) . \end{aligned}$$
  In this case, the mean curvature of the hypersurface $\{t\}\times \Sigma _{\kappa ,\beta }$ is given by
  23 $$\begin{aligned} H_{\kappa ,\beta }(t)= &   (n-1) \frac{f_{\kappa ,\beta }'(t)}{f_{\kappa ,\beta }(t)}\nonumber \\= &   (n-1) \sqrt{|\kappa |}\coth \left( \sqrt{|\kappa |}t + \text {arcoth}\left( \frac{\beta }{(n-1)\sqrt{|\kappa |}}\right) \right) \end{aligned}$$

Our first result shows that given a lower Ricci bound and a bound on the mean curvature of the Cauchy surface $\Sigma$, the area element of the evolving surface can be given an upper bound corresponding essentially to the area element in the respective model geometry.

### Lemma 3.2

*(Forward infinitesimal area comparison).* For $(t,\textbf{x}) \in \mathcal {D}^+$ the infinitesimal area $\mathcal {A}(t,x)$ satisfies

24 $$\begin{aligned} \mathcal {A}(t,\textbf{x}) \le \left( \frac{f_{\kappa ,H(\textbf{x})}(t)}{f_{\kappa ,H(\textbf{x})}(0)}\right) ^{n-1} \cdot \mathcal {A}(0,x)\le \left( \frac{f_{\kappa ,\beta }(t)}{f_{\kappa ,\beta }(0)}\right) ^{n-1}, \end{aligned}$$

where $H(\textbf{x})$ denotes the forward mean curvature of $\Sigma$ at $\textbf{x}$ (i.e. the mean curvature with respect to the future normal $\textbf{n}_{\textbf{x}}$) and $f_{\kappa ,\beta }$ is the comparison warping function of Remark 3.1.

### Proof

The second inequality is obvious since $H\le \beta$. In the following, denote by $\mathcal {S}_{A,t}$ the set $\mathcal {S}_t \cap \exp _{\Sigma }^+((\{t\} \times A)\cap \mathcal {D}^+)$ for $A \subseteq \Sigma$. By [38, Thm. 8, Eq. (15)] for any measurable $A \subseteq \Sigma$,

25 $$\begin{aligned} \frac{\sigma _t(\mathcal {S}_{A,t})}{\sigma (A)} \le \left( \frac{f_{\kappa ,\tilde{\beta }}(t)}{f_{\kappa ,\tilde{\beta }}(0)}\right) ^{n-1}, \end{aligned}$$

where $\tilde{\beta }$ is any upper bound of *H* on *A* and $\sigma _t$ is the Riemannian measure on $\mathcal {S}_t$. The proof of Lemma 2.7 shows that if $\{t\} \times A \subseteq \mathcal {D}^+$, then

26 $$\begin{aligned} \sigma _t(\mathcal {S}_{A,t}) = \int _A \mathcal {A}(t,\textbf{x}) \, d\sigma (\textbf{x}). \end{aligned}$$

Now suppose $(t,\textbf{x}) \in \mathcal {D}^+$. Choose a sequence of open, relatively compact $\Sigma$-balls $U_i$ around $\textbf{x}$ that collapse to $\{ \textbf{x}\}$, then eventually $\{t\} \times U_i \subseteq \mathcal {D}^+$ and by continuity

27 $$\begin{aligned} \sigma _t(\mathcal {S}_{U_i,t}) \overset{i \rightarrow \infty }{\longrightarrow }\ \mathcal {A}(t,\textbf{x}), \end{aligned}$$

which, together with the comparison result referenced above (taking $\tilde{\beta }_i = \max _{\textbf{x}\in \overline{U}_i} H(\textbf{x})$), proves the claim. $\square$

### Remark 3.3

1. The proof of Lemma 3.2 is based on a result in [38, Thm. 8], which relies on $\Sigma$ being a Cauchy surface (or at least future causally complete) in order to conclude that any point in the evolution $\mathcal {S}_{A,t}$ is reached by a maximizing timelike geodesic from *A*. However, this is the only place where $\Sigma$ being Cauchy is necessary. If we restrict ourselves to $(t,\textbf{x})$ such that there exists an open neighborhood *U* of $\textbf{x}$ in $\Sigma$ and an $\epsilon >0$ such that $\exp ^+_{\Sigma }|_{(-\epsilon , t+\epsilon )\times U}$ is a diffeomorphism onto an open subset of *M*, then the argument goes through without assuming that $\Sigma$ is Cauchy. This will be relevant when applying the previous lemma in proving our backwards comparison result Lemma 3.4, as it will be applied to a level set in the evolution of $\Sigma$, which does not have to be a Cauchy surface in general.
2. Note that, if $\kappa <0$ and $\beta \ge -(n-1)\sqrt{|\kappa |}$, then $f_{\kappa ,\beta }$ first monotonically decreases to some minimum before monotonically increasing (cf. Remark 3.1), i.e., we get for $t \le T$
  28 $$\begin{aligned} \mathcal {A}(t,\textbf{x}) \le \max \left\{ 1, \left( \frac{f_{\kappa ,\beta }(T)}{f_{\kappa ,\beta }(0)}\right) ^{n-1}\right\} =:C^{A_+}(n,\kappa ,\beta ,T). \end{aligned}$$
3. If $\kappa < 0$ and $\beta < -(n-1)\sqrt{|\kappa |}$, then the model geometry is future timelike geodesically incomplete (cf. Remark 3.1) and a standard generalization of Hawking’s singularity theorem shows that (*M*, *g*) is future timelike geodesically incomplete (see, e.g., [20, Thm. 4.2] or [19]). Hence, we will mostly be interested in the case $\kappa ,\beta < 0$ and $\beta \ge -(n-1)\sqrt{|\kappa |}$.

In order to apply the area comparison results and prove a segment type inequality, we need to be able to estimate the area element of $\mathcal {S}_t$ from below. This is done in the next lemma and uses what we call *backwards comparison*. This simply means that instead of estimating the area element from above in terms of $\kappa$ and the mean curvature of $\Sigma$, we use the argument backwards (time reversed) from the evolution of $\Sigma$ at time *T*. As we still have control over the mean curvature at that time, we are able to estimate the area. Importantly, this procedure will result in a (non-trivial) bound on the area element from *below* in terms of $\kappa$, $\eta$, and *T*.

For the following, let $H:\mathcal {M}^+ \rightarrow \mathbb {R}$ denote the map assigning to each $p\in \mathcal {M}^+$ the future mean curvature of the smooth spacelike hypersurface $\mathcal {S}_{\tau _{\Sigma }(p)}$ at the point *p*. Clearly, $H|_{\Sigma }$ is then the mean curvature of $\Sigma$; hence, there is no confusion in denoting both by the same symbol *H*.

### Lemma 3.4

(Backward infinitesimal area comparison). For all $T,\eta > 0$, there is a constant $C^{A-}=C^{A-}(n,\kappa ,T,\eta )$ such that for all $t \in [0,T]$ and all $\textbf{x}\in {{\,\mathrm{Reg^+_{\eta }}\,}}(T)$,

29 $$\begin{aligned} \mathcal {A}(t,\textbf{x}) \ge \left( \frac{f_{\kappa ,-H(\exp _{\textbf{x}}(t \textbf{n}_{\textbf{x}}))}(0)}{f_{\kappa ,-H(\exp _{\textbf{x}}(t \textbf{n}_{\textbf{x}}))}(t)}\right) ^{n-1} \ge \left( \frac{f_{\kappa ,\tilde{\beta }(n,\kappa , \eta )}(0)}{f_{\kappa ,\tilde{\beta }(n,\kappa , \eta )}(T)}\right) ^{n-1}=:C^{A-}(n,\kappa , T, \eta ), \end{aligned}$$

where $$\tilde{\beta }(n, \kappa , \eta ):= (n-1) \sqrt{|\kappa |} \coth (\eta \sqrt{|\kappa |}) $$.

### Proof

The first inequality follows from changing the time orientation and running the argument of Lemma 3.2 backwards starting at $\exp _{\textbf{x}}(t \, \textbf{n}_{\textbf{x}})$.

It remains to argue that $-H(\exp _{\textbf{x}}(t \textbf{n}_{\textbf{x}}))\le (n-1) \sqrt{|\kappa |} \coth (\eta \sqrt{|\kappa |})$ for any $\textbf{x}\in {{\,\mathrm{Reg^+_{\eta }}\,}}(T)$. Assume to the contrary that

30 $$\begin{aligned} -H(\exp _{\textbf{x}}(t \textbf{n}_{\textbf{x}}))> (n-1) \sqrt{|\kappa |} \coth (\eta \sqrt{|\kappa |}), \end{aligned}$$

then by continuity of $\coth$ there is $\varepsilon > 0$ with $\varepsilon < \eta$ such that this inequality holds with $\eta$ replaced by $\eta - \varepsilon$, i.e., $H(\exp _{\textbf{x}}(t \textbf{n}_{\textbf{x}}))<- (n-1) \sqrt{|\kappa |} \coth ((\eta - \varepsilon ) \sqrt{|\kappa |})$, for some $\textbf{x}\in {{\,\mathrm{Reg^+_{\eta }}\,}}(T)$. Then, the future timelike unit speed geodesic starting at $\exp _{\textbf{x}}(t \textbf{n}_{\textbf{x}})$ and orthogonally to $\mathcal {S}_t$ is given by $s\mapsto \exp _{\textbf{x}}((t+s) \textbf{n}_{\textbf{x}})$. Note that this geodesic maximizes the distance to $\mathcal {S}_t$ for all $s\in [0,\eta ]$. By standard (forward) mean curvature/d’Alembertian comparison starting from $\mathcal {S}_t$ (cf. [38, Thm. 7]), $H(\exp _{\textbf{x}}((t+s) \textbf{n}_{\textbf{x}})) \le H_{\kappa , H(\exp _{\textbf{x}}(t \textbf{n}_{\textbf{x}}))}(s)$,^1^ for all $s\in [0,\eta ]$ and since $H(\exp _{\textbf{x}}(t \textbf{n}_{\textbf{x}}))<- (n-1) \sqrt{|\kappa |} \coth ((\eta - \varepsilon ) \sqrt{|\kappa |})=:\beta _0$, actually even

31 $$\begin{aligned} H(\exp _{\textbf{x}}((t+s) \textbf{n}_{\textbf{x}})) \le H_{\kappa , \beta _0}(s). \end{aligned}$$

Note that $\beta _0<-(n-1) \sqrt{|\kappa |}$, so checking with Remark 3.1, specifically equation (23),

32 $$\begin{aligned} H_{\kappa ,\beta _0 }=(n-1)\sqrt{|\kappa |}\coth (\sqrt{|\kappa |}s+ \coth ^{-1}\left( \frac{\beta _0}{(n-1)\sqrt{|\kappa |}}\right) ), \end{aligned}$$

so the previous inequality becomes

33 $$\begin{aligned} H(\exp _{\textbf{x}}((t+s) \textbf{n}_{\textbf{x}})) \le (n-1)\sqrt{|\kappa |}\coth (\sqrt{|\kappa |}s-(\eta - \varepsilon )\sqrt{|\kappa |}) . \end{aligned}$$

Now we see that the right-hand side diverges to $-\infty$ as $s\uparrow (\eta - \varepsilon ) < \eta$, but the left-hand site must remain finite for all $s\in [0,\eta )$ since $t\mapsto \exp _{\textbf{x}}(t \textbf{n}_{\textbf{x}})$ doesn’t have any $\Sigma$-cut points before $T+\eta$. $\square$

Closely connected to the evolution of the area element is the d’Alembertian of the time separation function $\tau _{\Sigma }$. Heuristically speaking, it measures the expansion of the volume spanned by neighboring geodesics of the congruence and usually comparison results for the d’Alembertian are established first and then used to show area comparison results. However, as we are basing our arguments upon already established comparison results we can treat both cases independently. As was the case for the area, it allows for an estimation from above with respect to the constant curvature comparison space which we include and which is shown in [38, Thm. 7]. The lower bound again follows from a backwards comparison argument.

### Lemma 3.5

*(Forward d’Alembert comparison).* For $(t,\textbf{x}) \in \mathcal {D}^+$, we have that

34 $$\begin{aligned} \Box _g \tau _{\Sigma }(\exp _{\Sigma }^+(t,\textbf{x}))= H(\exp _{\Sigma }^+(t,x)) \le H_{\kappa ,\beta }(t). \end{aligned}$$

### Lemma 3.6

*(Backward d’Alembert comparison).* For any $T,\eta > 0$, $t \in [0,T]$ and $\textbf{x}\in {{\,\mathrm{Reg^+_{\eta }}\,}}(T)$, we have that

35 $$\begin{aligned} \Box _g \tau _{\mathcal {S}_T}(\exp _{\Sigma }^+(t,\textbf{x})) = -H(\exp _{\Sigma }^+(t,x)) \le (n-1)\sqrt{|\kappa |}\coth (\eta \sqrt{|\kappa |}), \end{aligned}$$

where $\tau _{\mathcal {S}_T}$ is the time separation from the spacelike hypersurface $\mathcal {S}_T$ measured backwards.

### Proof

This was shown in the proof of Lemma 3.4. $\square$

### Remark 3.7

(On the comparison constants).

As already stated in the third point of Remark 3.3, we are mainly interested in the case $0 > \beta \ge -(n-1)\sqrt{|\kappa |}$, so

36 $$\begin{aligned} 0 < \frac{|\beta |}{(n-1)\sqrt{|\kappa |}} \le 1. \end{aligned}$$

For convenience, we first exclude the case $\beta = -(n-1)\sqrt{|\kappa |}$ and treat it separately later. While this case won’t be particularly relevant in our applications as we may replace any bound $\beta <0$ on *H* from above with some slightly bigger bound $\tilde{\beta } < 0$, including it does not pose any difficulties. Checking with Remark 3.1 then yields

37 $$\begin{aligned} f_{\kappa ,\beta }(t) = \frac{1}{\sqrt{|\kappa |}} \cosh \left( \sqrt{|\kappa |}t + \text {artanh}\left( \frac{\beta }{(n-1)\sqrt{|\kappa |}}\right) \right) . \end{aligned}$$

Hence, after some manipulations, the forward infinitesimal area comparison constant $C^{A+}=C^{A+}(n,\kappa ,\beta ,T)$ can be written as

38 $$\begin{aligned} C^{A+}(n,\kappa ,\beta ,T)&= \left( \frac{f_{\kappa ,\beta }(T)}{f_{\kappa ,\beta }(0)}\right) ^{n-1}\nonumber \\&= \left( \frac{\beta }{(n-1)\sqrt{|\kappa |}} \sinh (\sqrt{|\kappa |}T) + \cosh (\sqrt{|\kappa |}T)\right) ^{n-1}. \end{aligned}$$

Next, we want to derive an explicit form of the backward infinitesimal area comparison constant $C^{A-} = C^{A-}(n,\kappa ,T,\eta )$, cf. Lemma 3.4. First note that

39 $$\begin{aligned} \frac{\tilde{\beta }(n,\kappa ,\eta )}{(n-1)\sqrt{|\kappa |}} = \coth \left( \eta \sqrt{|\kappa |}\right) > 1 \end{aligned}$$

because $\coth (x) > 1$ for any $x > 0$. Again checking with Remark 3.1, the relevant function in this case is

40 $$\begin{aligned} f_{\kappa ,\tilde{\beta }}(t) = \frac{1}{\sqrt{|\kappa |}} \sinh \left( \sqrt{|\kappa |}t + \textrm{arcoth} \left( \frac{\tilde{\beta }}{(n-1)\sqrt{|\kappa |}}\right) \right) . \end{aligned}$$

Inserting $\tilde{\beta }=(n-1)\sqrt{|\kappa |} \coth \left( \eta \sqrt{|\kappa |}\right)$, we get

41 $$\begin{aligned} C^{A-}(n,\kappa ,T,\eta ) = \left( \frac{f_{\kappa ,\tilde{\beta }}(0)}{f_{\kappa ,\tilde{\beta }}(T)}\right) ^{n-1} = \left( \frac{\sinh (\eta \sqrt{|\kappa |})}{\sinh (\sqrt{|\kappa |}(T + \eta ))}\right) ^{n-1}. \end{aligned}$$

Next we consider the forward d’Alembert comparison constant $C^{\square +} = C^{\square +}(n,\kappa ,\beta ,T)$, by which we mean a uniform upper bound on the quantity $H_{\kappa ,\beta }(t)$ appearing in Lemma 3.5. By monotonicity of the comparison function $H_{\kappa ,\beta }$, we get

42 $$\begin{aligned} H_{\kappa , \beta }(t) \le H_{\kappa , \beta }(T)&= (n-1) \sqrt{|\kappa |}\tanh \left( \sqrt{|\kappa |}T + \text {artanh}\left( \frac{\beta }{(n-1)\sqrt{|\kappa |}}\right) \right) \end{aligned}$$

43 $$\begin{aligned}&= \frac{(n-1) \sqrt{|\kappa |}\tanh (\sqrt{|\kappa |}T)+\beta }{1+\tanh (\sqrt{|\kappa |}T) \frac{\beta }{(n-1)\sqrt{|\kappa |}} } =:C^{\square +}. \end{aligned}$$

The backward d’Alembert comparison constant $C^{\square -} = C^{\square -}(n,\kappa ,\eta )$ is already given in Lemma 3.6, namely

44 $$\begin{aligned} C^{\square -} = (n-1)\sqrt{|\kappa |}\coth (\eta \sqrt{|\kappa |}). \end{aligned}$$

Note that, contrary to $C^{A+},C^{A-}$ and $C^{\square -}$ which are always strictly positive, $C^{\square +}$ can be negative, zero, or positive depending on $n,\kappa , \beta ,T$.

We now briefly consider $\beta = -(n-1)\sqrt{|\kappa |}$: Clearly the expressions derived for $C^{A+}(n,\kappa ,\beta ,T)$ and $C^{\square +}(n,\kappa ,\beta ,T)$ above continuously extend to $\beta = -(n-1)\sqrt{|\kappa |}$, where they become $\exp (-\sqrt{|\kappa |}T)^{n-1}$ and $-(n-1)\sqrt{|\kappa |}$, respectively, which coincides with the bounds one gets when retracing the above steps for the model geometry in the $\beta = -(n-1)\sqrt{|\kappa |}$ case, where $f_{\kappa ,\beta }(t)=\exp (-\sqrt{|\kappa |}t)$ (cf. Remark 3.1).

### Remark 3.8

(On the d’Alembert operator).

Consider the diffeomorphism $\exp _{\Sigma }^+:\mathcal {D}^+ \rightarrow \mathcal {M}^+$. We have already noted that the metric *g* splits in $\exp _{\Sigma }^+$-coordinates, i.e.

45 $$\begin{aligned} (\exp _{\Sigma }^+)^*g = -dt^2 + g_t, \end{aligned}$$

where $g_t$ is the restriction of *g* to the spacelike hypersurface $\mathcal {S}_t \subseteq \mathcal {M}^+$. We choose coordinates $x = (x^j)$ on $\Sigma$ and thus also coordinates $(t,x^j) = (y^{\mu })$ on $\mathcal {M}^+$. Now suppose *F* is a smooth function on $\mathcal {M}^+$. We write $\dot{F}=\partial _t F$. Then

46 $$\begin{aligned} \Box _g (F^2)&= -\frac{1}{\sqrt{|\det g|}} \frac{\partial }{\partial y^{\mu }}\left( g^{\mu \nu } \sqrt{|\det g} \frac{\partial F^2}{\partial y^{\nu }}\right) \nonumber \\&= -\frac{1}{\sqrt{|\det g|}} \frac{\partial }{\partial t} \left( \underbrace{g^{00}}_{=-1} \underbrace{\sqrt{|\det g|}}_{=\sqrt{\det g_t}} \frac{\partial F^2}{\partial t}\right) \nonumber \\&\quad - \frac{1}{\sqrt{|\det g|}} \frac{\partial }{\partial x^i} \left( g_t^{ij} \sqrt{|\det g|} \frac{\partial F^2}{\partial x^j}\right) \nonumber \\&= 2\dot{F}^2 + 2F\ddot{F} + \frac{\partial _t \sqrt{\det g_t}}{\sqrt{\det g_t}} 2F\dot{F} - \Delta _{g_t}(F^2). \end{aligned}$$

For the time separation, we have that

47 $$\begin{aligned} \tau _{\Sigma }(\exp _{\Sigma }^+(t,\textbf{x})) = t. \end{aligned}$$

One then immediately checks (using the previous calculation, (45) and Remark 2.8) that

48 $$\begin{aligned} \Box _g (\tau _{\Sigma } \circ \exp _{\Sigma }^+) = \frac{\partial _t \sqrt{\det g_t}}{\sqrt{\det g_t}} = \frac{\partial _t \mathcal {A}}{\mathcal {A}}. \end{aligned}$$

Similarly, the backward time separation $\tau _{\mathcal {S}_T}$ on the *T*-slice satisfies (for $t \le T$)

49 $$\begin{aligned} \tau _{\mathcal {S}_T}(\exp _{\Sigma }(t,\textbf{x})) = T - t. \end{aligned}$$

As before,

50 $$\begin{aligned} \Box _g(\tau _{\mathcal {S}_T} \circ \exp _{\Sigma }) = -\frac{\partial _t \sqrt{\det g_t}}{\sqrt{\det g_t}} = - \frac{\partial _t \mathcal {A}}{\mathcal {A}}. \end{aligned}$$

Hence, d’Alembert comparison yields

51 $$\begin{aligned} \left| \frac{\partial _t \mathcal {A}}{\mathcal {A}} \right| = \left| \frac{\partial _t \sqrt{\det g_t}}{\sqrt{\det g_t}} \right| \le \max (C^{\square -},|C^{\square +}|) = C^{\square -}=:C^{\square }_{\max } \end{aligned}$$

on $$\Omega _T^+({{\,\mathrm{Reg^+_{\eta }}\,}}(T))$$.

Using the comparison results from the previous section, one can derive estimates for integrals along geodesics from estimates on volume integrals. The approach taken is similar in spirit to the segment inequality used in the almost splitting theorem by Cheeger–Colding ([5]; see also [34]). This technique is then used to obtain Ricci bounds along single geodesics from world volume inequalities, i.e., certain curvature integrals over a spacetime volume. To that end, we define a map $\mathcal {F}^T_f$ assigning to a point $\textbf{x}$ on $\Sigma$ the integral of a nonnegative *f* over the future-directed normal geodesic to $\Sigma$ starting at $\textbf{x}$ up to a time *T*.

### Definition 3.9

Let $f \ge 0$ be any nonnegative, continuous function on *M* and $T > 0$. Define

52 $$\begin{aligned} \mathcal {F}_f^T: \Sigma \rightarrow [0,\infty ],\quad \mathcal {F}^T_f(\textbf{x}):=\int _0^{\min (T,s^+(\textbf{x}))} f(\exp _{\Sigma }^+(t,\textbf{x})) dt. \end{aligned}$$

Next, we give a segment type inequality, i.e., a result inferring bounds on certain finite line integrals of *f* from a region $B\subseteq {{\,\mathrm{Reg^+_{\eta }}\,}}$ from bounds on the integral of *f* over the “tube” $\Omega _T^+(B)$.

### Proposition 3.10

*(Segment type inequality).* Let *f* be any continuous, nonnegative function on *M* and $T,\eta > 0$. Then for any measurable subset $B \subseteq {{\,\mathrm{Reg^+_{\eta }}\,}}(T)$ such that $0< \sigma (B) < \infty$ we have that

53 $$\begin{aligned} \inf _{\textbf{x}\in B} \mathcal {F}^T_f \le \frac{1}{C^{A-} \sigma (B)} \int _{\Omega _T^+(B)} f \, dvol_g, \end{aligned}$$

where $C^{A-}= C^{A-}(n,\kappa ,T,\eta )$ is the backward area comparison constant and $\sigma$ is the (Riemannian) volume measure on $\Sigma$.

### Proof

Using everything discussed so far in a straightforward calculation:

54 $$\begin{aligned} \int _{\Omega _T^+(B)} f \, dvol_g&= \int _{B} \int _0^T f(\exp _{\Sigma }^+(t,\textbf{x})) \mathcal {A}(t,\textbf{x}) dt d\sigma (\textbf{x}) \nonumber \\&\ge C^{A-} \int _{B}\int _0^T f(\exp _{\Sigma }^+(t,\textbf{x})) dt d\sigma (\textbf{x}) \nonumber \\&\ge C^{A-} \sigma (B) \inf _{\textbf{x}\in B} \mathcal {F}^T_f. \end{aligned}$$

$$\square $$

## The Singularity Theorems

We are now ready to present a typical singularity theorem which can be proved using the techniques we have developed. We decompose a function *f* into a sum of its positive and negative parts: $f = f_+ + f_-$ where $f_+ \ge 0$ and $f_- \le 0$ and write ${{\,\textrm{Ric}\,}}_-(U,U)$ for the negative part of $p\mapsto {{\,\textrm{Ric}\,}}(U_p,U_p)$.

We shall first state a singularity theorem adjacent result which has the advantage of following very directly from Proposition 3.10.

### Theorem 4.1

Let (*M*, *g*) be a globally hyperbolic spacetime with a smooth, spacelike Cauchy surface $\Sigma \subseteq M$ such that ${{\,\textrm{Ric}\,}}(v,v) \ge n\kappa$ for all unit timelike $v \in TM$ and $H \le \beta$, where $\kappa ,\beta < 0$, $\beta \ge -(n-1)\sqrt{|\kappa |}$. Let $B\subseteq \Sigma$ with $0<\sigma (B)< \infty$. If

55 $$\begin{aligned} \frac{1}{\sigma (B)} \int _{\Omega _T^+(B)} |{{\,\textrm{Ric}\,}}_-(U_p,U_p)| \, dvol_g(p) \le C^{A-}(n,\kappa ,\eta ,T) K, \end{aligned}$$

for any $T>0,\eta>0, K>0$ satisfying $K<|\beta |-\frac{n-1}{T}$, then $B \not \subseteq {{\,\mathrm{Reg^+_{\eta }}\,}}(T)$.

### Proof

Assume to the contrary that $B \subseteq {{\,\mathrm{Reg^+_{\eta }}\,}}(T)$. Set $f(p):=|{{\,\textrm{Ric}\,}}_-(U_p,U_p)|$, then Proposition 3.10 and the assumed estimate (55) imply that

56 $$\begin{aligned} \inf _{x \in B} \mathcal {F}_f^T \le K. \end{aligned}$$

Let $\varepsilon > 0$ be so small that $K + \varepsilon < |\beta |$, then there exists $\textbf{x}\in B$ such that

57 $$\begin{aligned} \mathcal {F}_f^T(\textbf{x})=\int _0^T f(\exp _{\textbf{x}}(t\textbf{n}_{\textbf{x}})) dt \le K + \varepsilon . \end{aligned}$$

Now the result follows directly from classical variational methods: Denoting by $c(t):=\exp _{\textbf{x}}(t \textbf{n}_{\textbf{x}})$ the normal geodesic starting in $\textbf{x}$, by assumption *c* maximizes the $\Sigma$-time separation up to $p:=\exp _{\textbf{x}}(T\textbf{n}_{\textbf{x}}) \in I^+(\Sigma )$. Let $E_i(t)$ be spacelike parallel orthonormal vector fields along *c* such that $\{\dot{c},E_1,\dots ,E_{n-1}\}$ is an orthonormal frame along *c*. Consider variations of *c* starting in $\Sigma$ and ending in *p* defined by variational vector fields $V_i(t):=(1-t/T)E_i(t)$. Denote by $L_i(t)$ the length functional of the corresponding variation. Using the second variation formula, it is a straightforward calculation (done e.g. in section 2 of [12] with a different sign convention) to arrive at

58 $$\begin{aligned} \sum _{i=1}^{n-1} L_i''(0)&= \left\langle \dot{c}(0), \sum _{i=1}^{n-1} II(E_i(0),E_i(0)) \right\rangle + \int _0^T \left( 1- \frac{t}{T}\right) ^2 {{\,\textrm{Ric}\,}}(\dot{c},\dot{c}) dt - \frac{n-1}{T}. \end{aligned}$$

Note that the first term on the right-hand side is equal to $-H(\textbf{x})$ (recall that we follow the conventions of [38]). Since *c* maximizes the $\Sigma$-time separation up to *p*, we get

59 $$\begin{aligned} 0 \ge \sum _{i=1}^{n-1} L_i''(0)&= -H(\textbf{x}) + \int _0^T \left( 1- \frac{t}{T}\right) ^2 {{\,\textrm{Ric}\,}}(\dot{c},\dot{c}) dt - \frac{n-1}{T} \nonumber \\&\ge |\beta | - \int _0^T \left( 1 - \frac{t}{T}\right) ^2 f(c(t)) dt - \frac{n-1}{T} \nonumber \\&\ge |\beta | - \int _0^T f(c(t))dt - \frac{n-1}{T} \nonumber \\&\ge |\beta | - K - \varepsilon - \frac{n-1}{T}, \end{aligned}$$

which implies that

60 $$\begin{aligned} T \le \frac{n-1}{|\beta | - K - \varepsilon }. \end{aligned}$$

Taking $\varepsilon \rightarrow 0$, we see that this contradicts our assumption on the relationship between *T*, *K*, and $|\beta |$. $\square$

While this is not a singularity theorem per se, as the conclusion of $B\not \subseteq {{\,\mathrm{Reg^+_{\eta }}\,}}$ only tells us that either a geodesic stops existing *or* maximizing the distance at or before $T+\eta$, its structure is very similar: Given the usual assumptions of singularity theorems (on ${{\,\textrm{Ric}\,}}$, causality, and the mean curvature of a Cauchy surface), it implies bounds on regions evolving from $\Sigma$ on which $\exp$ stays injective (even a diffeomorphism).

Let us now state a singularity theorem which still employs the segment inequality type methods, but assumes energy conditions inspired by QEIs. It will be used to prove geodesic incompleteness for the case of the non-minimally coupled classical scalar field in Sect. 5.

### Theorem 4.2

Let (*M*, *g*) be a smooth globally hyperbolic spacetime of dimension $n > 2$, $\Sigma \subseteq M$ a smooth, spacelike Cauchy surface. Suppose the following hold:

(i) There is $\kappa < 0$ such that ${{\,\textrm{Ric}\,}}(v,v) \ge n\kappa$ for all unit timelike $v \in TM$.
(ii) There exist $Q_1, Q_2 > 0$ such that for all $F \in C^{\infty }_c(\mathcal {M}^+)$, we have the following integral bound on the Ricci tensor:
  61 $$\begin{aligned} \int _{M} {{\,\textrm{Ric}\,}}(U_p,U_p) F(p)^2 \, dvol_g(p) \ge -Q_1\Vert F\Vert _{L^2(M)}^2 - Q_2 \Vert \dot{F}\Vert _{L^2(M)}^2. \end{aligned}$$
(iii) the mean curvature of $\Sigma$ satisfies
  62 $$\begin{aligned} -H \ge \min \left\{ (n-1)\sqrt{|\kappa |}\coth (\sqrt{|\kappa |}\tau ),\nu _*(n,\kappa , \tau ) \right\} \end{aligned}$$
  everywhere on $\Sigma$, where
  63 $$\begin{aligned}  &   \nu _*(n,\kappa ,\tau )\nonumber \\  &   \quad :=\min _{\{(T,\eta ): T+\eta =\tau \}}\min _{ \tau _0\in (0,T) } \bigg \{ \left( Q_1 + Q_2\frac{(C^{\square }_{\max }(n,\kappa , \eta ))^2}{4} + Q_2\frac{C^{\square }_{\max }(n,\kappa , \eta )}{2T}\right) \frac{T}{3} \nonumber \\  &   \qquad +Q_2 \left( 1+ \frac{TC^{\square }_{\max }(n,\kappa , \eta )}{2}\right) \left( \frac{1}{\tau _0}+\frac{1}{T-\tau _0}\right) + n |\kappa | \tau _0 \frac{2}{3} +\frac{n-1}{T-\tau _0} \bigg \} .\end{aligned}$$

Then no future-directed timelike curve emanating from $\Sigma$ has length greater than $\tau$ and (*M*, *g*) is future timelike geodesically incomplete.

### Proof

For $\min ((n-1)\sqrt{|\kappa |}\coth (\sqrt{|\kappa |}\tau ),\nu _*) =(n-1)\sqrt{|\kappa |}\coth (\sqrt{|\kappa |}\tau )$, this follows from classical comparison geometry techniques, cf. e.g. [20, Thm. 4.2] (note that $H\le (n-1)\sqrt{|\kappa |}\coth (-\sqrt{|\kappa |}\tau )$ implies $b_{\kappa ,\beta }=\tau$).

Let us therefore assume that $\min ((n-1)\sqrt{|\kappa |}\coth (\sqrt{|\kappa |}\tau ),\nu _*) =\nu _*$. Now assume for a contradiction that there is a future-directed timelike curve emanating from $\Sigma$ of length greater than $\tau =T+\eta$. Then ${{\,\mathrm{Reg^+_{\eta }}\,}}(T)$ is nonempty. Given any $f \in C^{\infty }_c({{\,\mathrm{\mathbb {R}}\,}})$ with support contained in [0, *T*], we pick an arbitrary, nonzero $h \in C^{\infty }_c(\Sigma )$ with support contained in ${{\,\mathrm{Reg^+_{\eta }}\,}}(T)$ and consider the function $F(t,\textbf{x}):=f(t)h(\textbf{x})(\mathcal {A}(t,\textbf{x}))^{-1/2}$ (in exponential coordinates). Then $\Vert F\Vert _{L^2(M)}^2 = \Vert f\Vert _{L^2({{\,\mathrm{\mathbb {R}}\,}})}^2 \Vert h\Vert _{L^2(\Sigma )}^2$ and

64 $$\begin{aligned} \dot{F}(t,\textbf{x}) = \dot{f}(t) h(\textbf{x}) \mathcal {A}(t,\textbf{x})^{-1/2} -\frac{1}{2} f(t)h(\textbf{x}) \mathcal {A}(t,\textbf{x})^{-3/2} \partial _t \mathcal {A}(t,\textbf{x}); \end{aligned}$$

hence,

65 $$\begin{aligned} \dot{F}(t,\textbf{x})^2&= \dot{f}(t)^2 h(\textbf{x})^2 \mathcal {A}(t,\textbf{x})^{-1} + \frac{1}{4} f(t)^2 h(\textbf{x})^2 \mathcal {A}(t,\textbf{x})^{-3} (\partial _t \mathcal {A}(t,\textbf{x}))^2 \nonumber \\&\phantom {aa}- \dot{f}(t) f(t) h(\textbf{x})^2 \mathcal {A}(t,\textbf{x})^{-2} \partial _t \mathcal {A}(t,\textbf{x}). \end{aligned}$$

By d’Alembert comparison, we can estimate

$$\begin{aligned} |\partial _t \mathcal {A} \cdot \mathcal {A}^{-1}|\le &   C^{\square }_{\max }(n,\kappa , \eta ):=\max (|C^{\square +}(n,\kappa , \eta )|,C^{\square -}(n,\kappa , \eta ))\\= &   C^{\square -}(n,\kappa , \eta ), \end{aligned}$$

as in Remark 3.8. Thus, dropping the dependence of $C^{\square }_{\max }$ on $n,\kappa ,\eta$ for now, and using the classical inequality

66 $$\begin{aligned} ab \le \varepsilon a^2 + \frac{1}{4 \varepsilon } b^2 , \end{aligned}$$

for $a,b \ge 0$ and $\varepsilon :=T/2$ we have

67 $$\begin{aligned} \Vert \dot{F}\Vert _{L^2(M)}^2\le &   \Vert \dot{f}\Vert _{L^2({{\,\mathrm{\mathbb {R}}\,}})}^2 \Vert h\Vert _{L^2(\Sigma )}^2 + \frac{(C^{\square }_{\max })^2}{4} \Vert f\Vert _{L^2({{\,\mathrm{\mathbb {R}}\,}})}^2 \Vert h\Vert _{L^2(\Sigma )}^2 \nonumber \\  &   + \frac{C^{\square }_{\max } }{2T}(T^2\Vert \dot{f}\Vert _{L^2({{\,\mathrm{\mathbb {R}}\,}})}^2+ \Vert f\Vert _{L^2({{\,\mathrm{\mathbb {R}}\,}})}^2)\Vert h\Vert _{L^2(\Sigma )}^2. \end{aligned}$$

On the other hand,

68 $$\begin{aligned}&\int _{\Omega _T^+({{\,\mathrm{Reg^+_{\eta }}\,}}(T))} {{\,\textrm{Ric}\,}}(U_p,U_p) F(p)^2 \, dvol_g(p)\nonumber \\&\quad \le \Vert h\Vert _{L^2(\Sigma )}^2 \sup _{\textbf{x}\in \textrm{supp}(h)} \int _0^T {{\,\textrm{Ric}\,}}(\dot{c}_{\textbf{x}}(t),\dot{c}_{\textbf{x}}(t)) f(t)^2 \, dt, \end{aligned}$$

where $\dot{c}_{\textbf{x}}(t)=U_{\exp _{\Sigma }^+(t,\textbf{x})}$. Since *h* has compact support in ${{\,\mathrm{Reg^+_{\eta }}\,}}(T)$, the supremum on the right-hand side is actually a maximum. Canceling the $\Vert h\Vert _{L^2(\Sigma )}^2$, by (61), we are left with

69 $$\begin{aligned}&\min _{\textbf{x}\in \textrm{supp}(h)} \left( - \int _0^T {{\,\textrm{Ric}\,}}(\dot{c}_{\textbf{x}}(t),\dot{c}_{\textbf{x}}(t)) f(t)^2 \, dt \right) \nonumber \\&\quad \le Q_1 \Vert f\Vert _{L^2({{\,\mathrm{\mathbb {R}}\,}})}^2\nonumber \\&\qquad + Q_2\left( \Vert \dot{f}\Vert _{L^2({{\,\mathrm{\mathbb {R}}\,}})}^2 +\frac{(C^{\square }_{\max })^2}{4} \Vert f\Vert _{L^2({{\,\mathrm{\mathbb {R}}\,}})}^2 + \frac{C^{\square }_{\max }}{2T} \left( T^2\Vert \dot{f}\Vert _{L^2({{\,\mathrm{\mathbb {R}}\,}})}^2 + \Vert f\Vert _{L^2({{\,\mathrm{\mathbb {R}}\,}})}^2\right) \right) \nonumber \\&\quad = \left( Q_1 + Q_2\frac{(C^{\square }_{\max })^2}{4} + Q_2\frac{C^{\square }_{\max }}{2T}\right) \Vert f\Vert _{L^2({{\,\mathrm{\mathbb {R}}\,}})}^2\nonumber \\&\qquad + Q_2 \left( 1+ \frac{TC^{\square }_{\max }}{2}\right) \Vert \dot{f}\Vert _{L^2({{\,\mathrm{\mathbb {R}}\,}})}^2. \end{aligned}$$

Fix $\textbf{x}_0\in \textrm{supp}(h)$ where the minimum is achieved and let $c:[0,T]\rightarrow M$ be the distance-to-$\Sigma$-maximizing geodesic $c_{\textbf{x}_0}$ starting orthogonally to $\Sigma$ at $\textbf{x}_0$.

Since *f* is still free (the only restriction is that its support is contained in [0, *T*]), we fix any $\tau _0 \in (0,T)$ and – following the definitions in [12, Lem. 4.1] for $m=1$ – choose $f(t)=\bar{f}(t)\phi (t)$ with

$$\begin{aligned} \bar{f}(t):={\left\{ \begin{array}{ll} 1\quad \quad \quad \quad \quad t\in [0,\tau _0]\\ 1-\frac{t-\tau _0}{T-\tau _0}\quad \quad t\in [\tau _0, T] \end{array}\right. } \end{aligned}$$

and

$$\begin{aligned} \phi (t):={\left\{ \begin{array}{ll} \frac{t}{\tau _0} \quad \quad t\in [0,\tau _0]\\ 1 \quad \quad t\in [\tau _0, T]. \end{array}\right. } \end{aligned}$$

With this choice, we closely follow the methods used in [12], especially Proposition 2.2 and partly Lemma 4.1 there, and will refer to that article for details of the index form methods applied. Note that *f* is piece-wise linear vanishing at 0 and *T*, so even though *f* is not smooth, all our above calculations, in particular (61) and (69), still hold (this is easily seen by approximating *f* with compactly supported smooth functions whose derivatives converge to the derivative of *f* in $L^2$). The next step is to estimate

$$\begin{aligned} J[\bar{f}]= \int _0^T \left( -{{\,\textrm{Ric}\,}}(\dot{c},\dot{c}) \bar{f}^2+(n-1)(\dot{\bar{f}})^2\right) dt \end{aligned}$$

from above (note the different sign convention for ${{\,\textrm{Ric}\,}}$ in [12]). Using the identity ${\bar{f}}^2= (\phi {\bar{f}})^2+(1-\phi ^2)$, we obtain:

70 $$\begin{aligned} J[\bar{f}]= &   \int _0^T -{{\,\textrm{Ric}\,}}(\dot{c},\dot{c}) \left( (\phi (t) \bar{f}(t))^2+(1-\phi (t)^2)\right) dt + (n-1) ||\dot{\bar{f}}(t)||^2 \nonumber \\\le &   \left( Q_1 + Q_2\frac{(C^{\square }_{\max })^2}{4} + Q_2\frac{C^{\square }_{\max }}{2T}\right) \Vert \phi \bar{f}\Vert _{L^2({{\,\mathrm{\mathbb {R}}\,}})}^2\nonumber \\  &   + Q_2 \left( 1+ \frac{TC^{\square }_{\max }}{2}\right) \left\| \frac{d}{dt}(\phi \, \bar{f})\right\| _{L^2({{\,\mathrm{\mathbb {R}}\,}})}^2 \nonumber \\    &   + n|\kappa | \int _0^{\tau _0} (1-\phi ^2) dt + \frac{n-1}{T-\tau _0} = \nonumber \\= &   \left( Q_1 + Q_2\frac{(C^{\square }_{\max })^2}{4} + Q_2\frac{C^{\square }_{\max }}{2T}\right) \frac{T}{3}+ Q_2 \left( 1+ \frac{TC^{\square }_{\max }}{2}\right) \left( \frac{1}{\tau _0}+\frac{1}{T-\tau _0}\right) \nonumber \\  &   + n|\kappa | \tau _0 \frac{2}{3} +\frac{n-1}{T-\tau _0}:=\nu _{\tau _0,\eta } , \end{aligned}$$

where we estimated ${{\,\textrm{Ric}\,}}(\dot{c}, \dot{c})\ge n \kappa$ and integrated the remaining terms involving $\phi$.

By [12, Lemma 4.1], there is a focal point along *c* if $J[\bar{f}]\le -H|_{\textbf{x}_0}$. Note that by (70) $J[\bar{f}]\le \nu _{\tau _0,\eta }$. Since this works for any $\tau _0\in (0,T)$ and any $\eta , T>0$ such that $T+\eta =\tau$, we may choose ${\tilde{\tau }}_0$ and ${\tilde{\eta }}+T$ such that $\nu _{{\tilde{\tau }}_0,{\tilde{\eta }}}$ becomes minimal (note that such a minimizing $\tau _0$ exists in the open interval because the expression goes to infinity as $\tau _0\rightarrow 0,T$). This leads to $J[\bar{f}] \le \nu _{{\tilde{\tau }}_0,{\tilde{\eta }}}= \nu _* \le -H|_{x_0}$ by the assumption (63), and there must be a focal point along *c* which in turn cannot be maximizing up to time $\tau =T+\eta$, contradicting $\textbf{x}_0\in {{\,\mathrm{Reg^+_{\eta }}\,}}(T)$. $\square$

### Remark 4.3

1. The proof of Theorem 4.2 really only needs the integral bound (61) for all $F\in C_c^\infty (\Omega _T^+({{\,\mathrm{Reg^+_{\eta }}\,}}(T)))$. However, as that set is in general hard to control, we chose to formulate Theorem 4.2 as is.
2. In the formulation of Theorem 4.2, higher derivatives of *F* could be accommodated for at the price of imposing bounds on the positive and negative parts individually (writing a function *b* as $b=b^+ + b^-$, $b^+ \ge 0$, $b^- \le 0$), i.e., with bounds of the form ($Q_+,Q_-,Q_+^{(m)},Q_-^{(m)} > 0$)
  71 $$\begin{aligned}&\int _{\Omega _T^+({{\,\mathrm{Reg^+_{\eta }}\,}}(T))} {{\,\textrm{Ric}\,}}_+(U_p,U_p) F(p)^2 \, dvol_g(p)\nonumber \\&\quad \ge Q_+ \Vert F\Vert _{L^2(M)}^2 + Q_+^{(m)} \Vert F^{(m)}\Vert _{L^2(M)}^2, \end{aligned}$$
  72 $$\begin{aligned}&\int _{\Omega _T^+({{\,\mathrm{Reg^+_{\eta }}\,}}(T))} |{{\,\textrm{Ric}\,}}_-(U_p,U_p)| F(p)^2 \, dvol_g(p)\nonumber \\&\quad \le Q_- \Vert F\Vert _{L^2(M)}^2 + Q_-^{(m)} \Vert F^{(m)}\Vert _{L^2(M)}^2. \end{aligned}$$
  Then, one can proceed as above to prove the existence of a singularity: Indeed, choosing *F* to be of the form $F(t,\textbf{x}) = f(t) h(\textbf{x})$ as before—just without the extra factor of $\mathcal {A}(t,\textbf{x})^{-\frac{1}{2}}$—one estimates $\int {{\,\textrm{Ric}\,}}_+ F^2 \, dvol_g$ forwards (using forward comparison) and backwards (using the assumption as well as backward comparison), and similarly for $\int {{\,\textrm{Ric}\,}}_- F^2 \, dvol_g$, to get an estimate on $\inf (-\int {{\,\textrm{Ric}\,}}f^2)$ as before. From these estimates, it is then clear by following [12] how $\nu _*$ has to be chosen, and the rest of the proof is completely analogous to Theorem 4.2.

## The Strong Energy Inequality

In this section, we want to show that the inequality (61) is satisfied by the non-minimally coupled classical scalar field, an example that violates the SEC. We additionally discuss the advantages of this bound compared to the worldline one.

### The Bound on the Effective Energy Density

This subsection is mainly based on results from [4]. The quantity of interest is the effective energy density (EED) given in Eq. (2). The field equation for non-minimally coupled scalar fields is

73 $$\begin{aligned} (\Box _g+m^2+\xi R)\phi =0 , \end{aligned}$$

where $\xi$ is the coupling constant and *R* is the Ricci scalar. The constant *m* has dimensions of inverse length, which would be the inverse Compton wavelength if one regarded (73) as the starting point for a quantum field theory with massive particles. The Lagrangian density is

74 $$\begin{aligned} L[\phi ]=\frac{1}{2} [(\nabla \phi )^2-(m^2+\xi R)\phi ^2 ] , \end{aligned}$$

where $(\nabla \phi )^2=-g^{\mu \nu } (\nabla _\mu \phi )(\nabla _\nu \phi )$. The stress energy tensor is obtained by varying the action with respect to the metric, giving

75 $$\begin{aligned} T_{\mu \nu }= &   (\nabla _\mu \phi )(\nabla _\nu \phi )-\frac{1}{2} g_{\mu \nu } (m^2 \phi ^2-(\nabla \phi )^2)\nonumber \\  &   +\xi (-g_{\mu \nu } \Box _g-\nabla _\mu \nabla _\nu +G_{\mu \nu }) \phi ^2 , \end{aligned}$$

where $G_{\mu \nu }$ is the Einstein tensor. We should observe here that the field equation and the Lagrangian reduce to those of minimal coupling for flat spacetimes, but the stress energy tensor does not.

The trace of the stress energy tensor is given by

76 $$\begin{aligned} T = \left( \frac{n}{2}-1\right) (\nabla \phi )^2 - \frac{n}{2}m^2\phi ^2 + \xi \left( -(n-1)\Box _g + \left( 1-\frac{n}{2}\right) R \right) \phi ^2 . \end{aligned}$$

We can write the stress energy tensor without mass dependence as

77 $$\begin{aligned} T_{\mu \nu }= &   (1-2\xi )(\nabla _\mu \phi )(\nabla _\nu \phi )+\frac{1}{2} (1-4\xi )( \phi \Box _g \phi +(\nabla \phi )^2)g_{\mu \nu }-2\xi \phi \nabla _\nu \nabla _\mu \phi \nonumber \\  &   \quad +\xi R_{\mu \nu } \phi ^2 -\frac{1}{2} g_{\mu \nu } (\phi P_\xi \phi ), \end{aligned}$$

where $P_\xi =\Box _g+m^2+\xi R$ is the Klein–Gordon operator. We also used the identity

78 $$\begin{aligned} \phi \Box _g \phi =\frac{1}{2} \Box _g \phi ^2-(\nabla \phi )^2 . \end{aligned}$$

Alternatively, we can remove the dependence on the D’Alembertian operator

79 $$\begin{aligned} T_{\mu \nu }= &   (1-2\xi )(\nabla _\mu \phi )(\nabla _\nu \phi )-\frac{1}{2} (1-4\xi )( m^2\phi ^2+\xi R \phi ^2-(\nabla \phi )^2)g_{\mu \nu } \nonumber \\  &   -2\xi \phi \nabla _\nu \nabla _\mu \phi +\xi R_{\mu \nu } \phi ^2 -2\xi g_{\mu \nu } (\phi P_\xi \phi ). \end{aligned}$$

Similarly for the trace, without explicit mass dependence we have

80 $$\begin{aligned} T=\left( \frac{n}{2}-1-2\xi (n-1)\right) (\nabla \phi )^2+\left( \frac{n}{2}-2\xi (n-1)\right) \phi \Box _g \phi +\xi R\phi ^2-\frac{n}{2} \phi P_\xi \phi ,\nonumber \\ \end{aligned}$$

while without dependence on the D’Alembertian operator

81 $$\begin{aligned} T= &   \left( \frac{n}{2}-1-2\xi (n-1)\right) (\nabla \phi )^2+\left( -\frac{n}{2}+2\xi (n-1)\right) m^2\phi ^2\nonumber \\  &   +\xi \left( 2\xi (n-1)+1-\frac{n}{2}\right) R\phi ^2 -2\xi (n-1) \phi P_\xi \phi . \end{aligned}$$

The EED without mass dependence is obtained by combining Eqs. (2), (77), and (80)

82 $$\begin{aligned} \rho _U= &   (1-2\xi )U^\mu U^\nu (\nabla _\mu \phi ) (\nabla _\nu \phi ) +\frac{1-2\xi }{n-2} \phi \Box _g\phi -\frac{2\xi }{n-2} (\nabla \phi )^2 \nonumber \\  &   -2\xi \phi U^\mu U^\nu \nabla _\mu \nabla _\nu \phi +\xi R_{\mu \nu } U^\mu U^\nu \phi ^2+\frac{\xi }{n-2}R\phi ^2-\frac{1}{n-2}\phi P_\xi \phi ,\nonumber \\ \end{aligned}$$

where we assumed $U^\mu$ is a unit timelike vector. We get the EED without dependence on the D’Alembertian by combining Eqs. (2), (79), and (81)

83 $$\begin{aligned} \rho _U= &   (1-2\xi ) U^\mu U^\nu (\nabla _\mu \phi )(\nabla _\nu \phi )-\frac{1-2\xi }{n-2} m^2 \phi ^2\nonumber \\  &   -\frac{2\xi }{n-2} (\nabla \phi )^2 -2\xi U^\mu U^\nu \phi \nabla _\mu \nabla _\nu \phi \nonumber \\  &   +\xi U^\mu U^\nu R_{\mu \nu } \phi ^2+\frac{2\xi ^2}{n-2} R \phi ^2-\frac{2\xi }{n-2} (\phi P_\xi \phi ) . \end{aligned}$$

The last term can be discarded “on shell,” i.e., for $\phi$ satisfying Eq. (73). For $\xi =0$, the EED further reduces to

84 $$\begin{aligned} \rho _U=U^\mu U^\nu (\nabla _\mu \phi )(\nabla _\nu \phi )+\frac{1}{n-2} \phi \Box _g \phi , \end{aligned}$$

or

85 $$\begin{aligned} \rho _U= U^\mu U^\nu (\nabla _\mu \phi )(\nabla _\nu \phi )-\frac{1}{n-2} m^2 \phi ^2 . \end{aligned}$$

We want to bound the following quantity where *x* is a spacetime variable, *F*(*x*) is a smearing function with compact support, and the integral is over a spacetime volume

86 $$\begin{aligned} \int dvol_g F^2(x) \rho _U . \end{aligned}$$

In [4], the authors derived two bounds for this quantity from Eqs. (82) and (83). Unlike the worldline case, for worldvolume averaging we can use successive integration by parts to derive a bound that has no explicit mass dependence and remains free from any field derivatives.

Introducing $V^\mu = F(x)U^\mu$, the averaged EED for the non-minimally coupled scalar field “on shell” obeys the following theorem [4, Thm. 2].

#### Theorem 5.1

If *M* is a manifold with metric *g* and dimension $n \ge 3$, $T_{\mu \nu }$ the stress energy tensor of a scalar field with coupling constant $\xi \in [0,\xi _v]$, and *f* a real-valued function on *M* with compact support, then “on shell ”

87 $$\begin{aligned} \int dvol_g \, \rho _U \, F^2(x) \ge -\min \{ \mathcal {B}_1,\mathcal {B}_2\} , \end{aligned}$$

where

88 $$\begin{aligned} \mathcal {B}_1= &   \int dVol \bigg \{ \frac{1-2\xi }{n-2} m^2 F^2(x) -\frac{2\xi ^2 R}{n-2} F^2(x) \nonumber \\  &   \qquad + \xi \left[ (\nabla _\mu V^\mu )^2+(\nabla _\mu V^\nu )(\nabla _\nu V^\mu )\right] \bigg \} \phi ^2 , \end{aligned}$$

and

89 $$\begin{aligned} \mathcal {B}_2= &   \int dvol_g \bigg \{ \frac{1-2\xi }{2(n-2)}(\Box _g F^2(x)) - \frac{\xi R}{n-2} F^2(x) \nonumber \\  &   \qquad +\xi [(\nabla _\mu V^\mu )^2+(\nabla _\mu V^\nu )(\nabla _\nu V^\mu )] \bigg \} \phi ^2 . \end{aligned}$$

Here

90 $$\begin{aligned} \xi _v= \frac{n-3}{2(n-2)} , \end{aligned}$$

where $\xi _v < 2\xi _c$ for any spacetime dimension $n>2$, while $\xi _c<\xi _v$ for $n\ge 4$. By $\xi _c$, we denote the conformal coupling in *n* dimensions

91 $$\begin{aligned} \xi _c=\frac{n-2}{4(n-1)} . \end{aligned}$$

For minimally coupled fields on any spacetime,

92 $$\begin{aligned} \mathcal {B}_1=\frac{1}{n-2} m^2 \int dvol_g \, F^2(x) \phi ^2 , \text { and } \mathcal {B}_2= \frac{1}{2(n-2)} \int dvol_g \, (\Box _g F^2(x)) \phi ^2 .\nonumber \\ \end{aligned}$$

For flat spacetimes, the bounds of Theorem 5.1 become

93 $$\begin{aligned} \mathcal {B}_1=\int dvol_g \left\{ \frac{1-2\xi }{n-2} m^2 F^2(x) +\xi [(\nabla _\mu V^\mu )^2+(\nabla _\mu V^\nu )(\nabla _\nu V^\mu )] \right\} \phi ^2 ,\nonumber \\ \end{aligned}$$

and

94 $$\begin{aligned} \mathcal {B}_2=\int dvol_g \bigg \{ \frac{1-2\xi }{2(n-2)} (\Box F^2(x)) + \xi [(\nabla _\mu V^\mu )^2+(\nabla _\mu V^\nu )(\nabla _\nu V^\mu )] \bigg \} \phi ^2 .\nonumber \\ \end{aligned}$$

In comparison, the worldline bound derived in [4] is

95 $$\begin{aligned} \int _\gamma dt \, \rho _U\, f^2(t)\ge &   - \int _\gamma dt \bigg \{ \frac{1-2\xi }{n-2} m^2 f^2(t)+\xi \bigg (2( f'(t))^2+R_{\mu \nu } \dot{\gamma }^\mu \dot{\gamma }^\nu f^2(t) \nonumber \\  &   \qquad - \frac{2\xi }{n-2} Rf^2(t) \bigg ) \bigg \}\phi ^2 , \end{aligned}$$

where the EED is averaged over a segment of a timelike geodesic $\gamma$. We note that only in the case of worldvolume averaging can we have a bound without mass dependence and field derivatives. That kind of bound has another important advantage which we can see by rescaling the support of the smearing function. We first let $\phi _{\text {max}}$ be the maximum amplitude of the field

96 $$\begin{aligned} \phi _{\text {max}}=\sup _M |\phi | , \end{aligned}$$

so we can take it out of the bound. Then we look at the bound $\mathcal {B}_2$ in flat spacetimes^2^

97 $$\begin{aligned} \int dvol_g \, \rho _U\, F^2(x)\ge &   - \phi _{\text {max}}^2 \int dvol_g \bigg \{ \frac{1-2\xi }{2(n-2)} (\left| \Box _g F^2(x) \right| ) \nonumber \\  &   \qquad + \xi [(\nabla _\mu V^\mu )^2+\left| (\nabla _\mu V^\nu )(\nabla _\nu V^\mu )\right| ] \bigg \} .\nonumber \\ \end{aligned}$$

Next, we consider a translationally invariant unit timelike vector field $U^\mu$ and define the rescaled smearing function $F_\lambda$ for $\lambda >0$ to be

98 $$\begin{aligned} F_\lambda (x)=\frac{F(x/\lambda )}{\lambda ^{n/2}} , \end{aligned}$$

so that its normalization is independent of the choice of $\lambda$. Then in the limit of large $\lambda$ the bound goes to zero and we have

99 $$\begin{aligned} \liminf _{\lambda \rightarrow \infty } \int dvol_g \, \rho _U\, F^2_\lambda (x) \ge 0 , \end{aligned}$$

thus establishing an averaged SEC (ASEC) for flat spacetimes. A similar calculation for the $\mathcal {B}_1$ bound gives a weaker, negative, bound in this case. The ASEC cannot be proved with the same assumptions for the worldline bound. In particular, for

100 $$\begin{aligned} \phi _{\text {max}}=\sup _\gamma |\phi | , \end{aligned}$$

and

101 $$\begin{aligned} f_{\lambda }(t)=\frac{f(t/\lambda )}{\sqrt{\lambda }} , \end{aligned}$$

we get

102 $$\begin{aligned} \liminf _{\lambda \rightarrow \infty } \int _\gamma dt \, \rho _U \, f^2_\lambda (t) \ge - \frac{1-2\xi }{n-2} m^2 \phi _{\text {max}}^2 , \end{aligned}$$

as shown in [4]. So for the wordline bound the long time average can be negative even for minimal coupling.

### The Curvature Bound

To derive a bound for the singularity theorem, we need to apply the Einstein equation

103 $$\begin{aligned} G_{\mu \nu }=8\pi T_{\mu \nu } . \end{aligned}$$

Taking the trace of the Einstein equation $G_{\mu \nu }=8\pi T_{\mu \nu }$ gives

104 $$\begin{aligned} \left( -\frac{n}{2}+1\right) R = 8\pi T . \end{aligned}$$

Using Eq. (81) “on shell” and using a similar method as [4], we can show

105 $$\begin{aligned} T= &   2(n-1)(\xi _c-\xi ) (\nabla \phi )^2 - \left( 1+2(n-1)(\xi _c-\xi ) \right) m^2\phi ^2 \nonumber \\  &   -2(n-1)(\xi _c-\xi )\xi R \phi ^2 . \end{aligned}$$

Using Eqs. (104) and (105), we have

106 $$\begin{aligned} \frac{n-2}{16\pi } \left( 1-8\pi \xi (1-\xi /\xi _c)\phi ^2\right) R= &   -2(n-1)(\xi _c-\xi ) (\nabla \phi )^2\nonumber \\  &   + \left( 1+2(n-1)(\xi _c-\xi ) \right) m^2\phi ^2 . \end{aligned}$$

Next, we assume that $8\pi \xi \phi ^2 \le 1/2$. Physically, it makes sense to consider values of $\phi ^2$ such that $8\pi \xi \phi ^2 \ll 1$ [16]. Considering $8\pi \xi \phi ^2$ close to 1 means considering field values close to the Planck scale, when our approach breaks down. Under that assumption we have $1-8\pi \xi (1-\xi /\xi _c)\phi ^2\ge 8\pi \xi \phi ^2$. Using this and the value of $\xi _c$ equation (106) yields

107 $$\begin{aligned} R\ge - \frac{ (\xi _c-\xi )}{\xi _c \xi \phi ^2} (\nabla \phi )^2 . \end{aligned}$$

Now we can replace the term including the Ricci scalar in the expression for EED of Eq. (82) ‘on shell’

108 $$\begin{aligned} \rho _U\ge &   (1-2\xi )U^\mu U^\nu (\nabla _\mu \phi ) (\nabla _\nu \phi ) +\frac{1-2\xi }{n-2} \phi \Box _g\phi \nonumber \\  &   -\frac{(\xi _c(1+2\xi )-\xi )}{\xi _c(n-2)} (\nabla \phi )^2 -2\xi \phi U^\mu U^\nu \nabla _\mu \nabla _\nu \phi \nonumber \\  &   +\xi R_{\mu \nu } U^\mu U^\nu \phi ^2, \end{aligned}$$

Using the Einstein equation and the definition of EED

109 $$\begin{aligned} 8\pi \rho _U= R_{\mu \nu } U^\mu U^\nu , \end{aligned}$$

we have the purely geometric bound

110 $$\begin{aligned} R_{\mu \nu }U^\mu U^\nu (1-8\pi \xi \phi ^2)\ge &   8\pi \bigg ((1-2\xi )U^\mu U^\nu (\nabla _\mu \phi ) (\nabla _\nu \phi ) +\frac{1-2\xi }{n-2} \phi \Box _g\phi \nonumber \\  &   -\frac{(\xi _c(1+2\xi )-\xi )}{\xi _c(n-2)} (\nabla \phi )^2-2\xi \phi U^\mu U^\nu \nabla _\mu \nabla _\nu \phi \bigg ).\nonumber \\ \end{aligned}$$

Using the identity of Eq. (78), we have

111 $$\begin{aligned} R_{\mu \nu }U^\mu U^\nu (1-8\pi \xi \phi ^2)\ge &   8\pi \bigg ((1-2\xi )U^\mu U^\nu (\nabla _\mu \phi ) (\nabla _\nu \phi ) -\frac{(2\xi _c-\xi )}{\xi _c(n-2)} (\nabla \phi )^2 \nonumber \\  &   +\frac{1-2\xi }{2(n-2)} \Box _g\phi ^2-2\xi \phi U^\mu U^\nu \nabla _\mu \nabla _\nu \phi \bigg ). \end{aligned}$$

Combining the first two terms gives

112 $$\begin{aligned} (1-2\xi )U^\mu U^\nu (\nabla _\mu \phi ) (\nabla _\nu \phi ) -\frac{(2\xi _c-\xi )}{\xi _c(n-2)} (\nabla \phi )^2= &   k(\xi ,n) U^\mu U^\nu (\nabla _\mu \phi )(\nabla _\nu \phi ) \nonumber \\  &   +\frac{(2\xi _c-\xi )}{\xi _c(n-2)}h^{\mu \nu }(\nabla _\mu \phi )(\nabla _\nu \phi ) ,\nonumber \\ \end{aligned}$$

where $h^{\mu \nu }=g^{\mu \nu }+U^\mu U^\nu$ is a positive semi-definite metric and

113 $$\begin{aligned} k(\xi ,n)=\frac{ \xi _c((n-4)-2\xi (n-2))+\xi }{\xi _c(n-2)} . \end{aligned}$$

Both terms in Eq. (112) are positive for $n>3$ as $k(\xi ,4)=\xi$ and $k(\xi ,n)>0$ for $n>4$ and $0\le \xi \le \xi _c$. Then, using the smearing function *F*(*x*) we can write

114 $$\begin{aligned}  &   \int dvol_g F(x)^2 R_{\mu \nu }U^\mu U^\nu (1-8\pi \xi \phi ^2) \nonumber \\  &   \quad \ge -8\pi \int dvol_g F(x)^2 \bigg (-\frac{1-2\xi }{2(n-2)} \Box _g\phi ^2+2\xi \phi U^\mu U^\nu \nabla _\mu \nabla _\nu \phi \bigg ). \end{aligned}$$

Integrating by parts the second term gives

115 $$\begin{aligned} 2\xi \int dvol_g F(x)^2 \phi U^\mu U^\nu \nabla _\mu \nabla _\nu \phi= &   -2\xi \int dvol_g F(x)^2(\nabla _U \phi )^2\nonumber \\  &   -2\xi \int dvol_g \nabla _U (F(x)^2)\phi \nabla _U \phi \nonumber \\  &   -2\xi \int dvol_g F(x)^2 \phi A^\nu \nabla _\nu \phi \nonumber \\  &   -2\xi \int dvol_g F(x)^2 \theta \phi (\nabla _U \phi ) , \end{aligned}$$

where we defined the expansion $\theta =\nabla _\mu U^\mu$ (note that this is $\textrm{tr}(\nabla U)$ in index-free notation). Here $\nabla _U U^\mu =A^\mu$ is the acceleration.

From now on, we will consider the geodesic flow emanating normally from the Cauchy surface $\Sigma$ as discussed in previous sections. Then the $U^\mu$ is tangent to the timelike geodesic parametrized by proper time *t*, $A=0$ and we have

116 $$\begin{aligned}  &   \int dvol_g F(x)^2 R_{\mu \nu }U^\mu U^\nu (1-8\pi \xi \phi ^2) \nonumber \\  &   \qquad \ge -8\pi \int dvol_g \bigg (\frac{1-2\xi }{2(n-2)}\phi ^2 \Box _g F(x)^2-2\xi \frac{\partial (F(x)^2)}{\partial t} \phi \dot{\phi }-2\xi F(x)^2 \theta \phi \dot{\phi } \bigg ).\nonumber \\ \end{aligned}$$

From Remark 3.8, we have

117 $$\begin{aligned} \Box _g F^2(x)= \frac{1}{\sqrt{\det g_t}} \partial _t(\sqrt{\det g_t} \partial _t (F(x)^2)) -\Delta _{g_t} F^2(x) . \end{aligned}$$

Replacing $\Box _g F^2(x)$ in Eq. (116), integrating by parts, and noting that $dvol_g=\sqrt{\det g_t} dtd\sigma (\textbf{x})$ give

118 $$\begin{aligned} \int dvol_g F(x)^2 R_{\mu \nu }U^\mu U^\nu (1-8\pi \xi \phi ^2)\ge &   -8\pi \int dvol_g \bigg (\frac{1-2\xi }{2(n-2)}F(x)^2 (-\Delta _{g_t} \phi ^2) \nonumber \\  &   -\frac{1}{(n-2)} 2 F(x) \dot{F}(x) \phi \,\dot{\phi }-2\xi F(x)^2 \theta \phi \dot{\phi } \bigg ) .\nonumber \\ \end{aligned}$$

Since $|\phi |<(8\pi \xi )^{1/2}$ we can absorb the factor $(1-8\pi \xi \phi ^2)^{1/2}$ in *F*(*x*)

119 $$\begin{aligned} \int dvol_g F(x)^2 R_{\mu \nu }U^\mu U^\nu\ge &   -8\pi \int dvol_g \bigg (\frac{1-2\xi }{2(n-2)}\frac{F(x)^2}{ (1-8\pi \xi \phi ^2)} (-\Delta _{g_t} \phi ^2) \nonumber \\  &   -\frac{2}{(n-2)} \frac{F(x) \dot{F}(x)}{ (1-8\pi \xi \phi ^2) } (\phi \,\dot{\phi })-2\xi \frac{F(x)^2}{(1-8\pi \xi \phi ^2)} \theta \phi \dot{\phi } \bigg ) ,\nonumber \\ \end{aligned}$$

where we omitted a strictly positive term. Using the following definitions

120 $$\begin{aligned} \dot{\phi }_{\max }=\sup _M \left| \frac{\partial }{\partial t}\phi \right| , \quad \Delta \phi _{\text {max}}^2=\sup \left| \Delta _{g_t} \phi ^2 \right| \end{aligned}$$

along with Eq. (96), we have

121 $$\begin{aligned}  &   \int dvol_g F(x)^2 R_{\mu \nu }U^\mu U^\nu \nonumber \\  &   \quad \ge -8\pi \int dvol_g \bigg \{ \frac{F(x)^2}{ (1-8\pi \xi \phi _{\text {max}}^2)} \bigg (\frac{1-2\xi }{2(n-2)}\Delta \phi _{\text {max}}^2 + \frac{\phi _{\text {max}}\,\dot{\phi }_{\max }}{T(n-2)}+2\xi |\theta | \phi _{\text {max}}\dot{\phi }_{\max }\bigg )\nonumber \\  &   \qquad +\frac{\dot{F}(x)^2}{ (1-8\pi \xi \phi _{\text {max}}^2) } \frac{T \phi _{\text {max}}\,\dot{\phi }_{\max }}{(n-2)} \bigg \} . \end{aligned}$$

Here we also used the inequality of Eq. (66).

### A Singularity Theorem for the Einstein–Klein–Gordon Theory

To proceed, we need to bound the absolute value of the expansion $\theta$. First we note that singularity theorems 4.2 or 4.3 use a function $F \in C^{\infty }_c(M)$ with support entirely contained in $\Omega _T^+({{\,\mathrm{Reg^+_{\eta }}\,}}(T))$ such that

122 $$\begin{aligned} (F \circ \exp _{\Sigma })(t, \textbf{x}) = f(t) h(\textbf{x}) \,, \end{aligned}$$

with $f \in C^{\infty }_c({{\,\mathrm{\mathbb {R}}\,}})$ with support contained in [0, *T*] and $h \in C^{\infty }_c(\Sigma )$ with support contained in ${{\,\mathrm{Reg^+_{\eta }}\,}}(T)$.

Now since $\theta (t,\textbf{x})=H(\exp _{\textbf{x}}(t \textbf{n}_{\textbf{x}}))$ on $\Omega _T^+({{\,\mathrm{Reg^+_{\eta }}\,}}(T))$, we can use Lemma 3.6 to obtain

123 $$\begin{aligned} \theta (t,\textbf{x}) {\ge } {-}(n-1)\sqrt{|\kappa |} \coth (\eta \sqrt{|\kappa |}){=}C^{\Box -} \quad \textrm{on}\;\Omega _T^+({{\,\mathrm{Reg^+_{\eta }}\,}}(T))\cong [0,T]{\times } {{\,\mathrm{Reg^+_{\eta }}\,}}(T).\nonumber \\ \end{aligned}$$

For an upper bound, we may use Lemma s 3.5 together with Remark 3.7 (as we assume $H\le \beta <0$ and we can always assume $\beta >-(n-1)\sqrt{|\kappa |}$ by making it smaller if necessary), i.e., standard mean curvature comparison, to obtain

124 $$\begin{aligned} \theta (t,\textbf{x})&=H((\exp _{\textbf{x}}(t \textbf{n}_{\textbf{x}}))) \le H_{\kappa , \beta }(T){=} C^{\Box +} \;\;\;\textrm{on}\;\Omega _T^+({{\,\mathrm{Reg^+_{\eta }}\,}}(T))\cong [0,T]\times {{\,\mathrm{Reg^+_{\eta }}\,}}(T).\nonumber \\ \end{aligned}$$

So, as in Remark 3.8,

125 $$\begin{aligned} |\theta (t,\textbf{x})|&\le \max \{C^{\Box -},|C^{\Box +}|\} = (n-1)\sqrt{|\kappa |} \coth (\eta \sqrt{|\kappa |}). \end{aligned}$$

From this, (121) becomes

126 $$\begin{aligned}  &   \int dVol \, R_{\mu \nu } U^\mu U^\nu \, F^2(x) \ge - Q_1 ||F||^2 -Q_2\Vert \dot{F}\Vert ^2 , \end{aligned}$$

for any $F \in C^{\infty }_c(M)$ with support entirely contained in $\Omega _T^+({{\,\mathrm{Reg^+_{\eta }}\,}}(T))$, where the constants are

127 $$\begin{aligned} Q_1= &   \frac{8\pi }{1-8\pi \xi \phi _{\text {max}}^2} \bigg [\frac{1-2\xi }{2(n-2)} \Delta \phi _{\text {max}}+\frac{\phi _{\text {max}}\dot{\phi }_{\max }}{T(n-2)}\nonumber \\  &   +2\xi (n-1)\sqrt{|\kappa |} \coth {(\eta \sqrt{|\kappa |})} \phi _{\text {max}}\dot{\phi }_{\max } \bigg ] , \end{aligned}$$

and

128 $$\begin{aligned} Q_2=\frac{8\pi }{1-8\pi \xi \phi _{\text {max}}^2} \frac{1}{n-2} T\phi _{\text {max}}\dot{\phi }_{\max } . \end{aligned}$$

We note that this bound is mass independent unlike the worldline bound derived in Ref. [4]. The appearance of spatial derivatives of the field can be considered small or negligible in some cases, for example, those of cosmological spacetimes. We note that in geometric units the constant $Q_1$ has dimensions of inverse time square while the constant $Q_2$ is dimensionless.

We are ready to state a singularity theorem for the non-minimally coupled classical scalar field.

#### Corollary 5.2

Let $(M, g, \phi )$ be a solution to the Einstein–Klein–Gordon equation in $n > 2$ spacetime dimensions. Let $(M, g, \phi )$ be globally hyperbolic with a smooth spacelike Cauchy surface $\Sigma$. Suppose $\phi$ has coupling $\xi \in [0, \xi _c]$. Assume hypotheses (i) and (iii) from Theorem 4.2 with constants $Q_1$ and $Q_2$ given by Eqs. (127) and (128). The constant $\phi _{\text {max}}$ is defined in (96) and obeys the inequality $8\pi \xi \phi _{\text {max}}^2 \le 1/2$. The constants $\dot{\phi }_{\max }$ and $\Delta \phi _{\text {max}}^2$ are defined as in (120). Then there exists a $\tau$ such that no future-directed timelike curve emanating from $\Sigma$ has length greater than $\tau$ and (*M*, *g*) is future timelike geodesically incomplete.

#### Proof

First we note that $(M,g,\phi )$ obeys the worldvolume bound of Eq. (126) for any $F \in C^{\infty }_c(M)$ with support entirely contained in $\Omega _T^+({{\,\mathrm{Reg^+_{\eta }}\,}}(T))$ (which is sufficient by Remark 4.3(1)). Thus, the condition (ii) of Theorem 4.2 is also obeyed with constants $Q_1$ and $Q_2$ given by Eqs. (127) and (128). The result follows from Theorem 4.2. $\square$

## Application

In this section, we estimate the required extrinsic curvature to have past timelike geodesic incompleteness for the non-minimally coupled scalar field in a simple cosmological model. The approach is based on the analysis performed in [4] and [12]. The results will be useful to compare our theorem with the worldline ones proven in the previous references.

One important estimate that needs to be made is the maximum allowed field value $\phi _{\text {max}}$. To do that, we follow Ref. [4] and estimate it using a hybrid model. The idea is to estimate the (classical) $\phi _{\text {max}}$ using the Wick square of a KMS state in Minkowski spacetime. That way we can connect the field value with a temperature *T*.

Assuming the scalar field describes an elementary particle with mass *m*, a maximum temperature where the model can be trusted is taken as much smaller than the Compton temperature $T_m=mc^2/k$ where *k* is the Boltzmann constant. Then $T_{\max }=10^{-2}T_m \ll T_m$. With these considerations, we have [4]

129 $$\begin{aligned} \phi _{\text {max}}^2\sim \langle {:}\phi ^2{:} \rangle _{T_{\max }}\sim 5\times 10^{-4} \frac{m^2 c^3}{\bar{h}} K_1(100) , \end{aligned}$$

where $K_x$ is the modified Bessel function.

On dimensional grounds, the ratio $\dot{\phi }_{\max }/\phi _{\text {max}}$ is proportional to $c/\lambda$ where $\lambda$ is taken as the Compton length of elementary particles. Then

130 $$\begin{aligned} \dot{\phi }_{\max }=\phi _{\text {max}}\frac{mc^2}{h} . \end{aligned}$$

Since we will consider a cosmological setting, we assume the field is spatially homogeneous and so $\Delta \phi _{\text {max}}^2=0$. The values of the field and its derivative only depend on the mass of an (elementary) particle. To compare our results with Refs. [4] and [12], we use the pion, the proton, and the Higgs. The pion has mass $2.40 \times 10^{-28}$kg, the proton $1.67 \times 10^{-27}$kg, and the Higgs $2.24 \times 10^{-25}$ kg.

Since we have been using geometric units, we need to restore the dimensions for this physical application. The quantity $C^{\square }_{\max }$ becomes

131 $$\begin{aligned} C^{\square }_{\max } = \max \{C^{\Box -},|C^{\Box +}|\} = 3\frac{\sqrt{G|\kappa |}}{c}\coth \left( \eta \frac{\sqrt{G|\kappa |}}{c}\right) , \end{aligned}$$

so it has dimensions of inverse time. Similarly, we restore dimensions in the constants $Q_1$ and $Q_2$. Both constants are multiplied by $G/c^4$, meaning $Q_2$ is dimensionless and $Q_1$ has dimensions of inverse time square.

After restoring the dimensions, we proceed with an estimation and optimization of the required extrinsic curvature $\nu _{\tau _0,\eta }$ of Theorem 4.2. First we optimize in terms of $\tau _0$ with the condition $T \gg \tau _0$. Then the optimal $\tau _0$, meaning the one that gives the minimum of $\nu _{\tau _0,\eta }$ is

132 $$\begin{aligned} \tau _0^{\text {opt}}=\sqrt{\frac{3}{2}}c\sqrt{\frac{Q_2(1+TC^{\square }_{\max }/2)}{G|\kappa |}} . \end{aligned}$$

To proceed, we will make some further approximations. First, for the rest of the calculation we assume $\xi =1/6$, the conformal coupling in 4-dimensions. In this case, any coupling in the allowed range is expected to give similar results. Then, we assume that

133 $$\begin{aligned} y \equiv \left( \eta \frac{\sqrt{G|\kappa |}}{c}\right) \ll 1 . \end{aligned}$$

With this assumption, we can take $\cosh {y} \approx 1$ and $\coth {y} \approx y^{-1}$. The maximum allowed value of $\eta$ so that the approximation is valid is $10^{-2} c/\sqrt{G\kappa }$. We fix the value of $\eta$ to be equal to that and we will revisit it to make sure $\eta \ll T$ as expected.

With these assumptions, for each particle, the required extrinsic curvature is a function of *T* and $\kappa$. As we expect $\eta \ll T$, we will refer to *T* as the maximum time to singularity. To compare the results of our theorem to cosmological data, we will use results from the PLANCK collaboration [1]. The Hubble parameter is the same as the extrinsic curvature of a cosmological spacelike Cauchy surface, and today it has an estimated value of $H_0=2.18 \times 10^{-18} s^{-1}$ while the age of the universe is $t_0=4.35 \times 10^{17}s$.^3^ The goal is to find the largest possible $\kappa$ so that $\nu \le H_0$ and *T* is as close as possible to $t_0$.

We note that $\nu$ has a term of the form $\sim 3/T$ similar to the worldline theorem of Ref. [12]. That means *T* has to be larger than $1.5 \times 10^{18} s$ to have the required extrinsic curvature smaller than the measured one. Considering that we want a prediction of the singularity time, we examine values of *T* between this value and $10^{20} s$, about three orders of magnitude larger than $t_0$. For each elementary particle and within this range of *T*, we found the maximum possible value of $\kappa$ for our theorem to apply. The results are presented in Table 1.

**Table 1:** Masses, maximum time to singularity, and required $\kappa$ to have past geodesic incompleteness for three elementary particles. We note that in all cases $\eta \ll T$

Particle	Mass in kg	*T* in *s*	$\kappa$ in $J/m^3$	$\eta$ in *s*
Pion	$2\times 10^{-28}$	$3.05 \times 10^{18}$	$5.18 \times 10^{20}$	16.18
Proton	$1.67 \times 10^{-27}$	$2.94 \times 10^{18}$	$1.07 \times 10^{19}$	112.52
Higgs	$2.23 \times 10^{-25}$	$3.06 \times 10^{18}$	$5.93 \times 10^{14}$	$1.51 \times 10^{4}$

Comparing our results with the ones from Ref. [12] where the same model was examined using a worldline inequality, we find several differences. First of all with the worldvolume inequality, we are able to predict past geodesic incompleteness for the Higgs which was impossible in the worldline case. Second, our method doesn’t require the SEC to hold near the Cauchy surface; thus, this assumption was dropped and we are able to apply the theorem using the spacelike cosmological Cauchy surface today. The drawback of our theorem is the requirement for a global pointwise condition. However, we find that the negative energy density allowed at each spacetime point is very large, comparable to the energy density at the very early universe.

## Discussion

In this work, we prove the first singularity theorems using a worldvolume inequality as an assumption. Using area comparison results in a Lorentzian manifold, we are able to derive bounds on individual timelike geodesics from worldvolume integrals. Additionally, using index form methods developed in [12] we prove a singularity theorem with a worldvolume integrated energy condition. Our results have physical applications, and as an example, we apply our theorem in the case of the classical non-minimally coupled field, which violates the strong energy condition. Finally, we apply our theorem using a toy cosmological model and compare our results with the ones from worldline inequalities. We find that the worldvolume theorem could give better results, especially in the case of elementary particles with large mass such as the Higgs boson.

The obvious extension of this work is to apply the theorem to a quantum field theory using a quantum energy inequality as an assumption as done in Ref. [13]. There are currently a few obstacles for this application: Theorem 4.2 is proved only for one timelike derivative on the smearing function while the quantum inequality requires two. Perhaps more importantly, the theorem has as an assumption a pointwise energy condition. Even though the negative energy allowed can be large, in the context of quantum field theory there are no lower energy bounds for spacetime points. Thus, the pointwise condition would need to be replaced with an integral one.

The null geodesic incompleteness case seems even more interesting than the timelike one. As it is impossible to bound the null energy on single geodesic segments [14], but it is possible on finite null surfaces [15], it would be of great interest to prove a Penrose-type theorem for worldvolume inequalities. One mathematical difficulty arising in the null case is that there are no direct Riemannian analogues to draw from; however, there are null area comparison results, cf. [6, 23], controlling the $(n-2)$-dimensional area of constant-affine-parameter slices of the lightcone. This work is currently in progress.

## Footnotes

1 As in Remark 3.1 here $H_{\kappa ,\beta }(t)$ is the mean curvature of the spacelike hypersurface $\{t\} \times \Sigma _{\kappa ,\beta }$ in the comparison geometry $(a_{\kappa ,\beta }b_{\kappa ,\beta }) \times _{f_{\kappa ,\beta }} (\Sigma _{\kappa ,\beta },h_{\kappa ,\beta })$.

2 This bound differs from the analogous one in [4] where the absolute values were incorrectly omitted.

3 There is an experimental ambiguity regarding the value of the Hubble constant. Here we use the value measured using the CMB perturbations.
